# UCP2 Upregulates ACSL3 to Enhance Lipid Droplet Release from Acinar Cells and Modulates the Sirt1/Smad3 Pathway to Promote Macrophage‐to‐Myofibroblast Transition in Chronic Pancreatitis

**DOI:** 10.1002/advs.202412571

**Published:** 2025-08-28

**Authors:** Kunpeng Wang, Lilong Zhang, Beiying Deng, Wanrong Jiang, Tianrui Kuang, Chen Chen, Kailiang Zhao, Qiao Shi, Jun He, Weixing Wang

**Affiliations:** ^1^ Department of General Surgery Renmin Hospital of Wuhan University Wuhan 430060 China; ^2^ General Surgery Laboratory Renmin Hospital of Wuhan University Wuhan 430060 China; ^3^ Hubei Key Laboratory of Digestive System Disease Wuhan 430060 China; ^4^ Department of Geriatric Renmin Hospital of Wuhan University Wuhan 430060 China; ^5^ Department of General Surgery The Second Xiangya Hospital Central South University Changsha Hunan 410011 China

**Keywords:** acinar cells, ACSL3, chronic pancreatitis, lipid droplet, macrophage‐to‐myofibroblast transition, Sirt1, Smad3, uncoupling protein 2

## Abstract

Chronic pancreatitis (CP) is a progressive inflammatory disease characterized by pancreatic fibrosis and functional decline. Here, we identify macrophage‐to‐myofibroblast transition (MMT) as a novel feature of CP and investigate the role of mitochondrial uncoupling protein 2 (UCP2) in this process. Using mouse models, human pancreatic specimens, and cell lines, we show that UCP2 is markedly upregulated in CP, primarily in acinar cells. UCP2 knockout reduces MMT and alleviates fibrosis, whereas macrophage depletion reverses this protective effect, confirming the central role of MMT. Metabolomic profiling reveals that UCP2 knockout alters lipid metabolism by downregulating acyl‐CoA synthetase long‐chain family member 3 (ACSL3) and reducing lipid droplet (LD) release in acinar cells. Mechanistically, UCP2 upregulation increases silent information regulator 1 (Sirt1) expression, enhances Smad3 phosphorylation and nuclear translocation, and activates transforming growth factor‐β (TGF‐β)/Smad3 signaling to promote macrophage MMT. Macrophage‐specific Sirt1 knockout suppresses both fibrosis and MMT. In conclusion, UCP2 drives CP progression by regulating ACSL3‐mediated LD release in acinar cells and modulating macrophage function through the Sirt1/Smad3 pathway. Targeting UCP2 may represent a promising therapeutic strategy for CP.

## Graphical abstract

These findings suggest that UCP2 promotes LD formation and release in acinar cells by upregulating ACSL3 expression. This alteration in the local lipid metabolic environment indirectly drives the MMT process. Additionally, UCP2 may regulate the acetylation of Smad3 through Sirt1, enhancing its nuclear activity and activating the TGF‐β/ Smad3 signaling pathway. This, in turn, promotes fatty acid metabolism, providing energy for macrophage MMT and synergistically inducing pancreatic fibrosis.

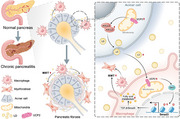

## 1 Introduction

Chronic pancreatitis (CP) is a progressive, irreversible disease with a rising global incidence in recent years.^[^ ^1^ ^]^ Its pathological features include acinar cell damage, immune cell infiltration, pancreatic atrophy, and interstitial fibrosis, which lead to both endocrine and exocrine pancreatic dysfunction.^[^ ^2^ ^]^ Consequently, CP can result in pancreatic diabetes, pancreatic calcification, ductal stones, and an increased risk of pancreatic cancer. Current clinical treatments are limited, primarily due to the inability to reverse pancreatic fibrosis.^[^ ^3^ ^]^

Uncoupling protein 2 (UCP2) primarily regulates oxidative stress and energy metabolism. It controls not only the number and secretion of insulin and glucagon from pancreatic α and β cells but also pancreatic development.^[^ ^4^ ^]^ Furthermore, UCP2 knockdown significantly inhibits the proliferation of pancreatic stellate cells (PSCs) in pancreatitis.^[^ ^5^ ^]^ Importantly, UCP2 mediates glutamine/aspartate metabolism, influencing pancreatic cancer progression.^[^ ^6^ ^]^ These findings suggest that UCP2 may be a crucial regulatory gene in pancreatic diseases; however, its role in CP remains unexplored.

Disorders of lipid metabolism are common features of CP,^[^ ^7^ ^]^ and lipid droplets (LD) are key cellular organelles involved in this process.^[^ ^8^ ^]^ Recent research highlights the role of LD formation and release in regulating cellular energy metabolism, protecting cells from external stressors, and influencing both health maintenance and disease progression.^[^ ^9^ ^]^ UCP2‐regulated mitochondrial uncoupling plays a crucial role in lipid metabolism. The influence of UCP2 on lipid synthesis and NADPH metabolism contributes to oxidative defense in colorectal cancer,^[^ ^10^ ^]^ while its regulation of lipid metabolism in macrophages impacts inflammatory responses.^[^ ^11^ ^]^ Additionally, LD‐mediated fatty acid metabolism determines the immune phenotype of macrophages.^[^ ^12^ ^]^ Macrophages, acinar cells, and PSCs are recognized as critical components of the fibrotic microenvironment in CP. Traditional views suggest that the persistent activation of PSCs is a primary driver of pancreatic fibrosis. However, the activation of PSCs does not directly cause CP fibrosis. Instead, the activation of myofibroblasts and extracellular matrix deposition are the key factors driving CP fibrosis. The role of macrophage polarization and the interactions between macrophages and PSCs in promoting pancreatic fibrosis have been well documented.^[^ ^13^ ^]^ However, due to the high degree of macrophage plasticity, it remains unclear whether macrophages consistently play a pro‐fibrotic role in CP, as well as the specific timing of their transformation. Macrophage‐to‐myofibroblast transition (MMT) is a recent discovery in fibrotic diseases,^[^ ^14^ ^]^ where macrophages transform into myofibroblasts. These myofibroblasts can comprise 30–65% of the total myofibroblast population and are pivotal in kidney fibrosis and the activation of tumor‐associated fibroblasts.^[^ ^15^, ^16^ ^]^ However, it remains unclear whether MMT occurs in pancreatic fibrosis and what mechanisms are involved, as these aspects have not yet been reported.

The aforementioned studies highlight the significant and critical roles of lipid metabolism and macrophage plasticity in CP fibrosis. UCP2 emerges as a key factor linking these two elements in the mediation of CP. Recognized as a crucial target for lipid metabolism regulation, UCP2 influences macrophage plasticity through its regulation of glycolysis and redox signaling.^[^ ^17^, ^18^ ^]^ Furthermore, the mechanism by which lipid accumulation in the pancreatic microenvironment drives CP progression via a cascade amplification effect, initiated by IL‐33‐induced type II immune responses, has been elucidated.^[^ ^7^ ^]^ While UCP2‐mediated uncoupling is vital for mitochondrial metabolic reprogramming and macrophage plasticity, particularly in the differentiation of macrophages in response to IL‐33 stimulation.^[^ ^19^ ^]^ Recent findings also reveal that acinar cells can activate PSCs through autophagy, further advancing the fibrosis process.^[^ ^20^ ^]^ However, the mechanisms by which pancreatic acinar cells influence macrophages to promote pancreatic fibrosis remain poorly understood. Additionally, determining whether UCP2‐regulated LD and MMT contribute to CP progression remains a crucial unresolved research question.

In this study, we identified UCP2 as a crucial regulatory gene in CP and established MMT as a novel feature of the disease. By using Alpha‐Smooth Muscle Actin ( α‐SMA) in combination with F4/80 or CD68 co‐staining in CP mouse models and patient samples, we were able to identify myofibroblasts derived from macrophages. Mechanistically, UCP2 upregulates ACSL3 in pancreatic acinar cells, which promotes LD release and initiates MMT in macrophages. Additionally, UCP2 directly regulates MMT through the Sirt1/Smad3 signaling pathway. Importantly, we demonstrated that UCP2 knockout and macrophage‐specific silencing of the Sirt1/Smad3 pathway block MMT and significantly inhibit CP progression. Therefore, UCP2‐regulated MMT may serve as a novel therapeutic target for CP.

## 2 Results

### 2.1 UCP2 Is Markedly Upregulated in CP, Predominantly Within Acinar Cells

**Figure 1:**
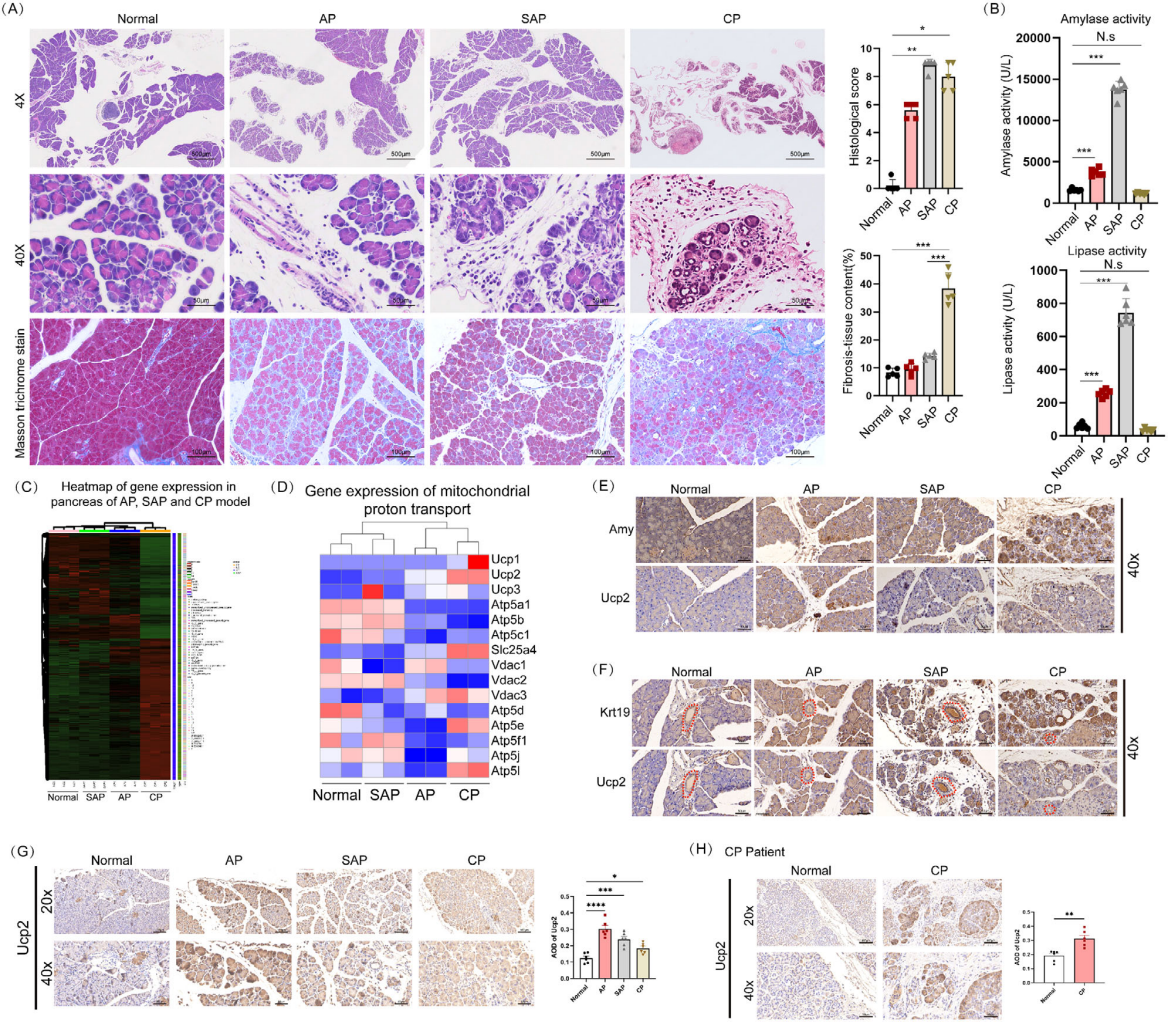
Upregulation of UCP2 in CP and its localization in acinar cells. A) HE staining of pancreatic tissues from normal, AP, SAP, and CP mouse models. 40x or 400x magnification, scale bar, 500 or 50 µm. *n* = 5. Masson staining reveals significantly increased fibrosis in CP compared to normal, AP, and SAP groups. 200x magnification, scale bar, 100 µm. *n* = 5. B) Serum levels of trypsin and pancreatic amylase were significantly elevated in the AP and SAP groups. *n* = 6. C,D) Transcriptome analysis identifies UCP family genes as potential key regulators in the transition from AP and SAP to CP. E,F) Sequential immunohistochemical results of Krt19 and Amy with UCP2 in pancreatic samples from the Normal, AP, SAP, and CP groups. The red staining indicates pancreatic ducts. The results suggest that UCP2 is more predominantly colocalized with acinar cells. 400x magnification, scale bar, 50 µm. G) Immunohistochemistry confirms significant UCP2 upregulation across AP, SAP, and CP models. 200x or 400x magnification, scale bar, 100 or 50 µm. *n* = 6. H) Immunohistochemical analysis of UCP2 expression in human CP samples, 200x or 400x magnification, scale bar, 100 or 50 µm. *n* = 6. Data are presented as the mean ± SEM from three independent experiments, ^*^ *p* < 0.05, ^**^ *p* < 0.01, ^***^ *p* < 0.001, and ^****^ *p* < 0.001, NS, no significant difference.

CP can arise from acute pancreatitis (AP), including its severe form, severe acute pancreatitis (SAP). To investigate the key genes driving this transition, we established mouse models of AP, SAP, and CP. Hematoxylin & eosin (HE) staining showed that, compared to the normal group, the AP model exhibited marked pancreatic edema and lobular disorganization. The SAP model exhibited widespread acinar necrosis and extensive inflammatory cell infiltration, surpassing the AP model. The CP model displayed acinar atrophy, pseudoductal formation, inflammatory infiltration, and extensive fibrosis (**Figure** **1**A). Pancreatic histological scoring confirmed significantly higher scores in the AP, SAP, and CP groups compared to the normal group (Figure 1B). Masson staining further demonstrated a significant increase in fibrotic area in the CP group compared to the control, AP, and SAP groups (Figure 1A,B). Trypsin and pancreatic amylase levels were markedly elevated in the AP and SAP groups compared to Normal, with the highest levels in the SAP group, over two/three times those in the AP group. However, no significant differences were detected between the CP and control groups (Figure 1B).

Further exploration of key genes involved in the progression from AP and SAP to CP, using transcriptome sequencing (Figure 1C), identified mitochondrial proton transport and especially the mitochondrial uncoupling proteins (UCPs) genes as potential key regulators. (Figure 1D). The UCP gene family consists of five distinct genes. Unlike UCP1, primarily expressed in brown adipose tissue, UCP3 is expressed in skeletal muscle and cardiac tissue, and UCP4 and UCP5 are expressed in brain tissue,^[^ ^21^ ^]^ UCP2 is widely expressed across various human tissues, including the pancreas, where it plays a crucial role in hormone secretion, as well as glucose and lipid metabolism.^[^ ^22^ ^]^ Moreover, UCP2 exhibits over 95% homology, compared to only 58% and 71% for UCP1 and UCP3, respectively.^[^ ^23^ ^]^ Thus, our study focuses on the significant role of UCP2 in CP.

The pancreatic parenchyma is primarily composed of acinar cells and ductal cells. Krt19 and amylase (Amy) were used to label ductal and acinar cells, respectively, and sequential immunohistochemistry was performed to determine the localization of UCP2 within pancreatic parenchymal cells. The results showed that UCP2 exhibited significant colocalization with acinar cells (Figure 1E), but not with ductal cells (Figure 1F). Furthermore, compared with the normal group, UCP2 expression was markedly upregulated in AP, SAP, and CP (Figure 1G). In human CP specimens, UCP2 expression was also increased compared with normal pancreatic tissue (Figure 1H). Taken together, these findings indicate that UCP2 is significantly upregulated in CP and is predominantly localized within acinar cells.

### 2.2 Knockdown of UCP2 Significantly Inhibits the Progression of CP in Mice

**Figure 2:**
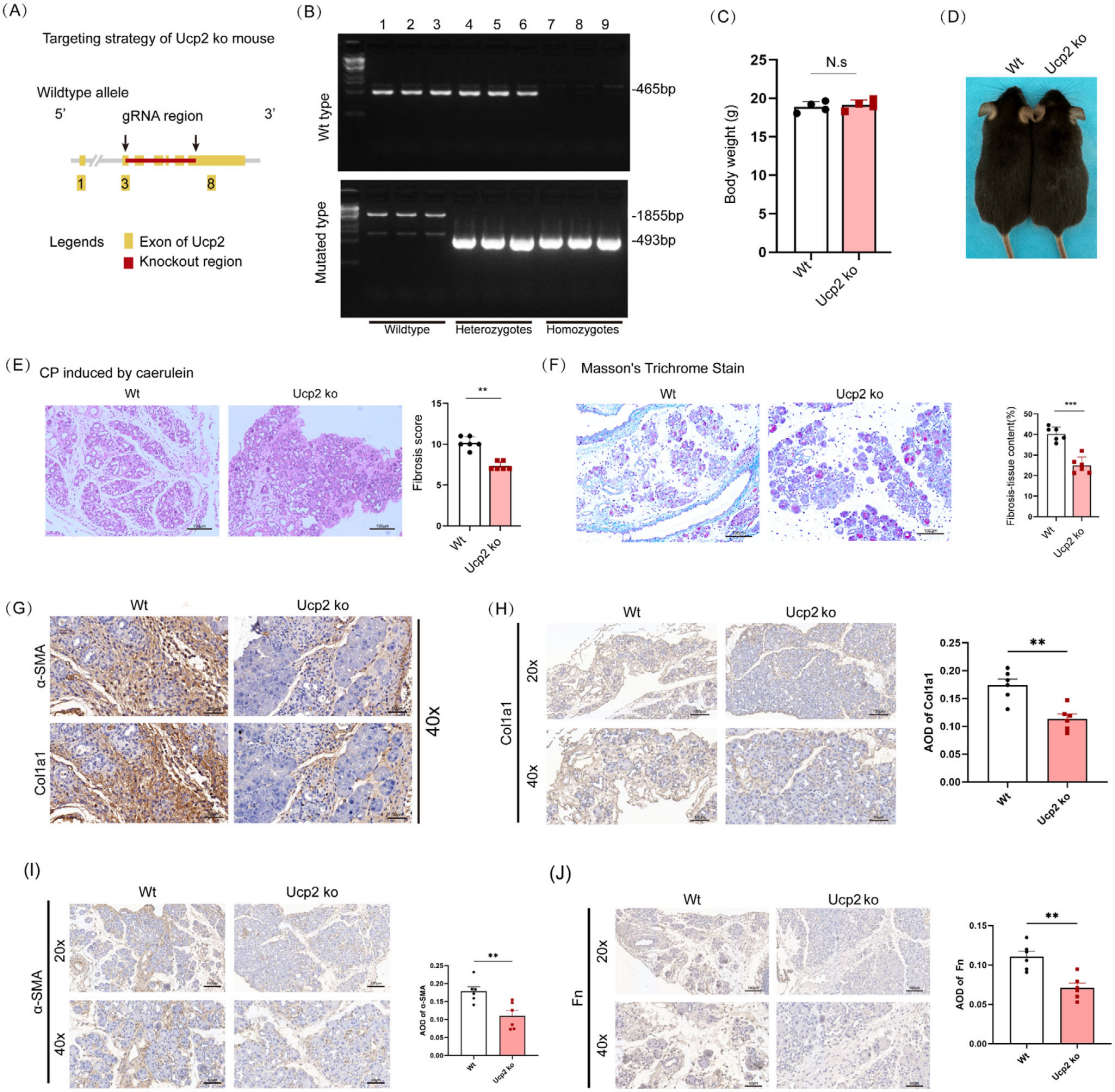
Impact of UCP2 knockdown on CP progression in mice. A) Confirmation of UCP2 knockout in UCP2‐KO mice through genotyping. B) Verification of UCP2 knockout via Agarose Gel Electrophoresis. C) Assessment of body weight gain in UCP2 KO and WT mice. *n* = 4. D) Measurement of body size in UCP2 KO mice, and WT mice. E) HE staining and fibrosis score of pancreatic tissues from UCP2‐KO and WT mice. 200x magnification, scale bar, 100 µm. *n* = 6. F) Masson's staining of pancreatic tissues reveals a marked reduction in fibrosis in UCP2‐KO mice relative to WT. 200x magnification, scale bar, 100 µm. *n* = 6. G) Sequential immunohistochemistry staining results of α‐SMA and Col1a1. 400x magnification, scale bar, 50 µm. H–J) Immunohistochemical results for Col1A1, α‐SMA, and Fn by UCP2 KO and WT Mice. 200x or 400x magnification, Scale bar, 100 or 50 µm. *n* = 6. Data are expressed as the mean ± SEM of three independent experiments. ^**^ *p* < 0.01, ^***^ *p* < 0.001, N.S, no statistical difference.

Given the potential role of UCP2 in CP, we successfully generated UCP2‐knockout (UCP2 KO) mice (**Figure** **2**A,B). UCP2 knockout had no impact on body weight gain (Figure 2C) or body size (Figure 2D). Previous studies suggest that global UCP2 knockout may accelerate pancreatic development during embryogenesis, resulting in an approximately twofold increase in pancreatic volume in UCP2 KO mice compared with Wild Type (WT) mice.^[^ ^4^ ^]^

To further explore the effect of UCP2 gene knockout on pancreatic development, we employed Amy to label acinar cells, Insulin to label β‐cells, and Krt19 to label duct cells. Various parameters were compared between age‐matched mice (1–6 weeks). Immunofluorescence analysis revealed that at 1 and 2 weeks of age, UCP2 KO mice exhibited significantly larger absolute surface areas of Amy‐, Insulin‐, and Krt19‐positive cells compared to WT mice. However, from 3 weeks onward, no significant differences were observed between the two groups (Figure S1, Supporting Information). HE staining conducted during weeks 1–6 showed no significant differences in the morphology or structure of the pancreas between age‐matched UCP2 KO and WT mice (Figure S2A, Supporting Information). Moreover, pancreatic weight did not vary significantly between the two groups (Figure S2B, Supporting Information). By week 6, no differences were observed in pancreatic elastase‐1 (FE‐1) levels, oral glucose tolerance test (OGTT) results, area under the curve (AUC), or extracellular matrix marker expression (Figure S2C–F, Supporting Information). These results indicate that UCP2 knockout does not impact basic pancreatic function in adult mice.

We then established a CP model in UCP2‐KO mice through continuous caerulein injections and observed that UCP2 knockout markedly reduced the mice's sensitivity to caerulein. This was evidenced by the attenuation of typical CP characteristics, including acinar atrophy, pseudotubular formation, inflammatory infiltration, and fibrosis. As a result, the fibrosis score was reduced (Figure 2E). Furthermore, fibrosis in the CP model was significantly alleviated following UCP2‐knockout, as confirmed by Masson's staining results (Figure 2F). To further elucidate the impact of UCP2 knockout on the expression of fibrosis‐related genes in acinar cells, we performed sequential immunohistochemistry for Collagen Type I Alpha 1 Chain (Col1a1) and α‐SMA, which revealed the tissue‐level localization patterns and differential expression of these two key fibrotic markers (Figure 2G). However, we recognized that co‐expression analysis in acinar cells alone may not fully reflect the overall extent of pancreatic fibrosis. Therefore, we further confirmed by immunohistochemistry that UCP2 knockout downregulated the expression levels of Col1 a1, α‐SMA, and Fn (Figure 2H–J). These findings suggest that UCP2‐knockout significantly inhibits the fibrotic process in CP.

### 2.3 MMT Represents a Novel Characteristic of CP, and UCP2 Knockout Suppresses Pancreatic Fibrosis by Inhibiting the MMT Process

α‐SMA is a widely recognized marker of activated myofibroblasts,^[^ ^14^ ^]^ and MMT has recently been identified as a novel feature of fibrotic diseases. Cells co‐expressing the myofibroblast marker α‐SMA along with macrophage markers such as F4/80 and CD68 are classified as MMT cells. Considering that UCP2 knockout significantly reduces α‐SMA expression, we aimed to investigate the presence of MMT in CP. We identified MMT cells in CP mice using immunofluorescence‐based combined staining.

**Figure 3:**
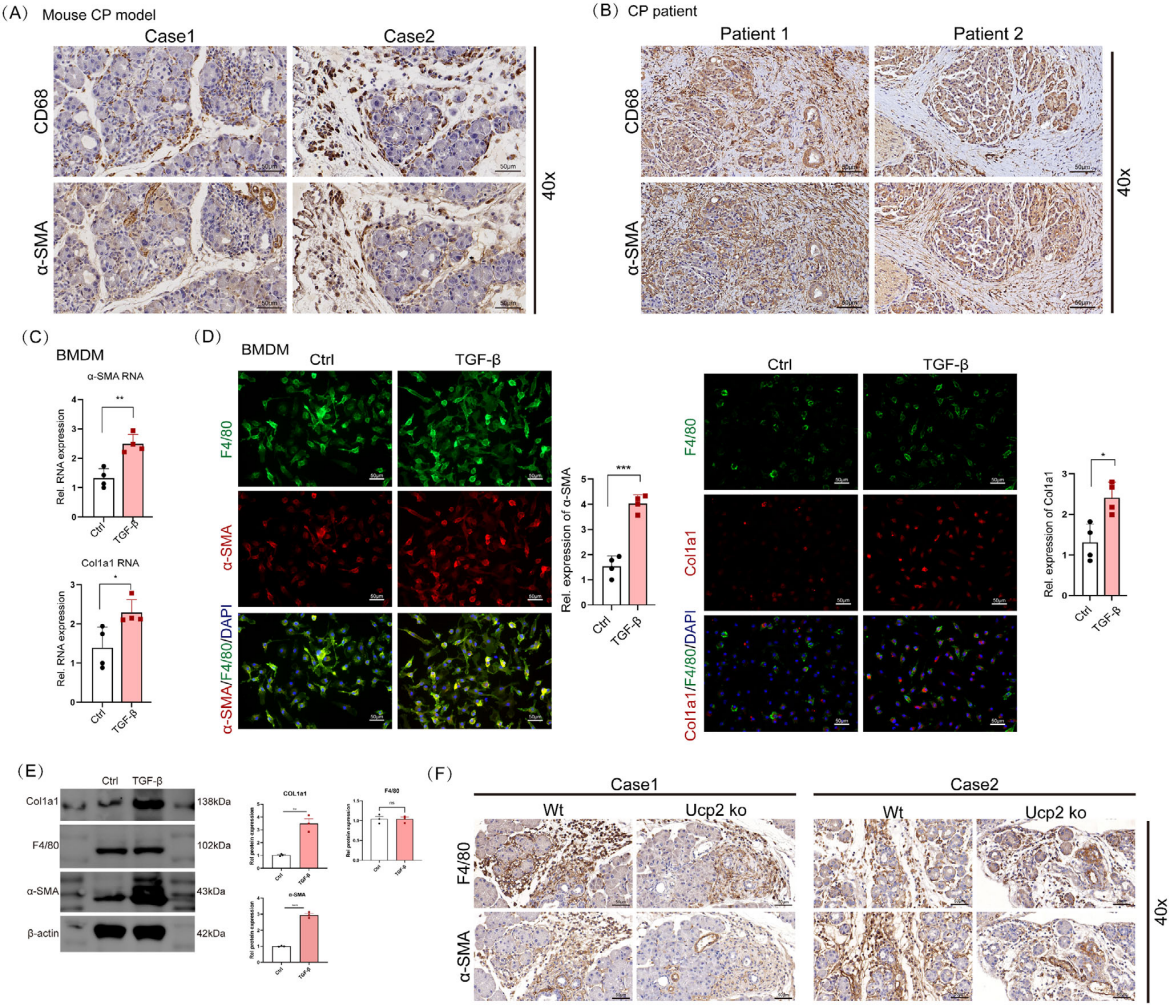
MMT is a novel feature of CP, and UCP2 knockdown significantly inhibited the MMT process in CP. A) Sequential immunohistochemistry analysis demonstrating colocalization of α‐SMA and CD68 in pancreatic tissues of CP mice. 400x magnification, scale bar, 50 µm. B) Similar colocalization pattern confirmed in human CP samples. 400x magnification, scale bar, 50 µm. C) qPCR analysis of BMDMs stimulated with TGF‐β reveals a significant increase in mRNA levels of fibrosis‐related genes α‐SMA and Col1a1, indicating successful induction of the MMT phenotype. *n* = 4. D) Immunofluorescence further demonstrates elevated expression levels of α‐SMA and Col1a1 in BMDMs following TGF‐β stimulation, confirming MMT induction in vitro. *n* = 4. E) WB showed that TGF‐β stimulation significantly upregulated the protein expression levels of Col1a1 and α‐SMA in BMDMs while the expression level of F4/80 showed no significant change. *n* = 3. F) Sequential immunohistochemistry analysis demonstrating that the colocalization of F4/80 and α‐SMA in pancreatic tissues was significantly decreased following UCP2 knockout. 400x magnification, scale bar, 50 µm. Data are expressed as the mean ± SEM of three independent experiments; ^*^ *p* < 0.05, ^**^ *p* < 0.01, and ^***^ *p* < 0.001.

Sequential immunohistochemistry revealed the colocalization of α‐SMA and CD68 in pancreatic tissues of CP mice (**Figure** **3**A), which was also confirmed in human CP samples (Figure 3B). Furthermore, multiplex immunofluorescence demonstrated that ≈30% of myofibroblasts in both CP mice and human samples originated from macrophages (Figure S3A,B, Supporting Information).

To investigate whether Transforming Growth Factor Beta (TGF‐β) alone could induce the transdifferentiation of bone marrow‐derived macrophages (BMDMs) into myofibroblasts in vitro, qPCR analysis revealed that TGF‐β stimulation significantly increased the mRNA expression levels of fibrosis‐related genes α‐SMA and Col1a1 (Figure 3C). Immunofluorescence analysis further confirmed that TGF‐β stimulation led to a substantial increase in the expression levels of α‐SMA and Col1a1 in BMDMs (Figure 3D). Additionally, WB confirmed a significant upregulation of Col1a1 and α‐SMA protein expression following TGF‐β stimulation, while the expression level of F4/80 showed no significant change (Figure 3E). These results demonstrate the successful induction of the MMT phenotype in macrophages in vitro.

To further investigate whether UCP2 influences the MMT process in CP, we examined the effects of UCP2 knockout on MMT. Sequential immunohistochemistry revealed that colocalization of F4/80 and α‐SMA was significantly reduced after UCP2 knockout (Figure 3F), while multiplex immunofluorescence indicated that the proportion of MMT cells in pancreatic tissue of CP mice decreased from ≈30% to ≈15% following UCP2 knockout (Figure S3C, Supporting Information).

**Figure 4:**
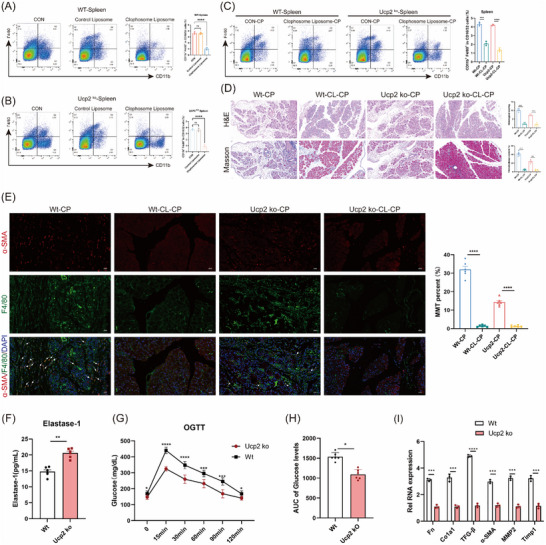
Effects of macrophage depletion and UCP2 knockout on pancreatic fibrosis and function in CP mice. A–C) Flow cytometry analysis confirmed that macrophage depletion reduced splenic macrophage levels in both WT and UCP2 KO mice (mean 3.56% versus 0.78%), with sustained depletion at the end of CP modeling (mean 4.26% versus 1.72%). *n* = 3. D) H&E and Masson's trichrome staining showed that macrophage depletion attenuated caerulein‐induced pathological features and fibrosis in CP. 200x magnification, Scale bar, 50 µm. *n* = 6. E) Multiplex immunofluorescence staining for α‐SMA and F4/80 revealed nearly absent MMT cells in both WT and UCP2 KO mice after macrophage depletion. 400x magnification, Scale bar, 50 µm. *n* = 6. F) UCP2 knockout increased FE‐1 expression in pancreatic tissue. *n* = 6. G,H) OGTT and AUC analyses showed improved glucose tolerance in CP mice following UCP2 knockout. *n* = 6. I) UCP2 knockdown reduced RNA expression of pancreatic extracellular matrix components. *n* = 3. Data are expressed as the mean ± SEM of three independent experiments; ^*^ *p* < 0.05, ^**^ *p* < 0.01, ^***^ *p* < 0.001, and ^****^ *p* < 0.001 NS, no statistical difference.

Although macrophage‐derived myofibroblasts constitute ≈30% of the total myofibroblast population, their precise role in CP remains unclear. To investigate their function, we administered an in vivo macrophage depletion agent to WT and UCP2 KO mice and assessed its effects on MMT and pancreatic fibrosis. Flow cytometry confirmed that macrophage depletion effectively reduced splenic macrophage levels in both WT and UCP2 KO mice (mean 3.56% vs 0.78%), whereas the liposome control group showed no significant effect (**Figure** **4**A,B). The depletion agent was administered every three days during CP induction, and splenic macrophage levels were reassessed at the end of CP modeling, confirming sustained depletion (mean 4.26% vs 1.72%) (Figure 4C).

HE and Masson's trichrome staining further demonstrated that macrophage depletion significantly attenuated caerulein‐induced pathological features and fibrosis in CP (Figure 4D). Multiplex immunofluorescence staining for α‐SMA and F4/80 revealed that pancreatic MMT cells were nearly absent in both WT and UCP2 KO mice following macrophage depletion (Figure 4E). These findings indicate that macrophage depletion disrupts MMT, thereby negating the antifibrotic effect of UCP2 knockout in CP.

CP is commonly associated with dysfunction in both the exocrine and endocrine components of the pancreas. To assess whether UCP2 knockout improves pancreatic function, we examined its effects on both systems. The results demonstrated that UCP2 knockout significantly increased FE‐1 expression in pancreatic tissue (Figure 4F). Furthermore, OGTT and AUC analyses confirmed that UCP2 knockout enhanced glucose tolerance in CP mice (Figure 4G,H). Additionally, UCP2 knockdown markedly reduced the RNA expression levels of pancreatic extracellular matrix components (Figure 4I). In conclusion, MMT represents a novel characteristic of CP, and UCP2 knockout suppresses pancreatic fibrosis by inhibiting the MMT process.

### 2.4 UCP2 Knockout does not Alter the Intrinsic Expression of Fibrosis‐Related Phenotype in Macrophages or PSCs

**Figure 5:**
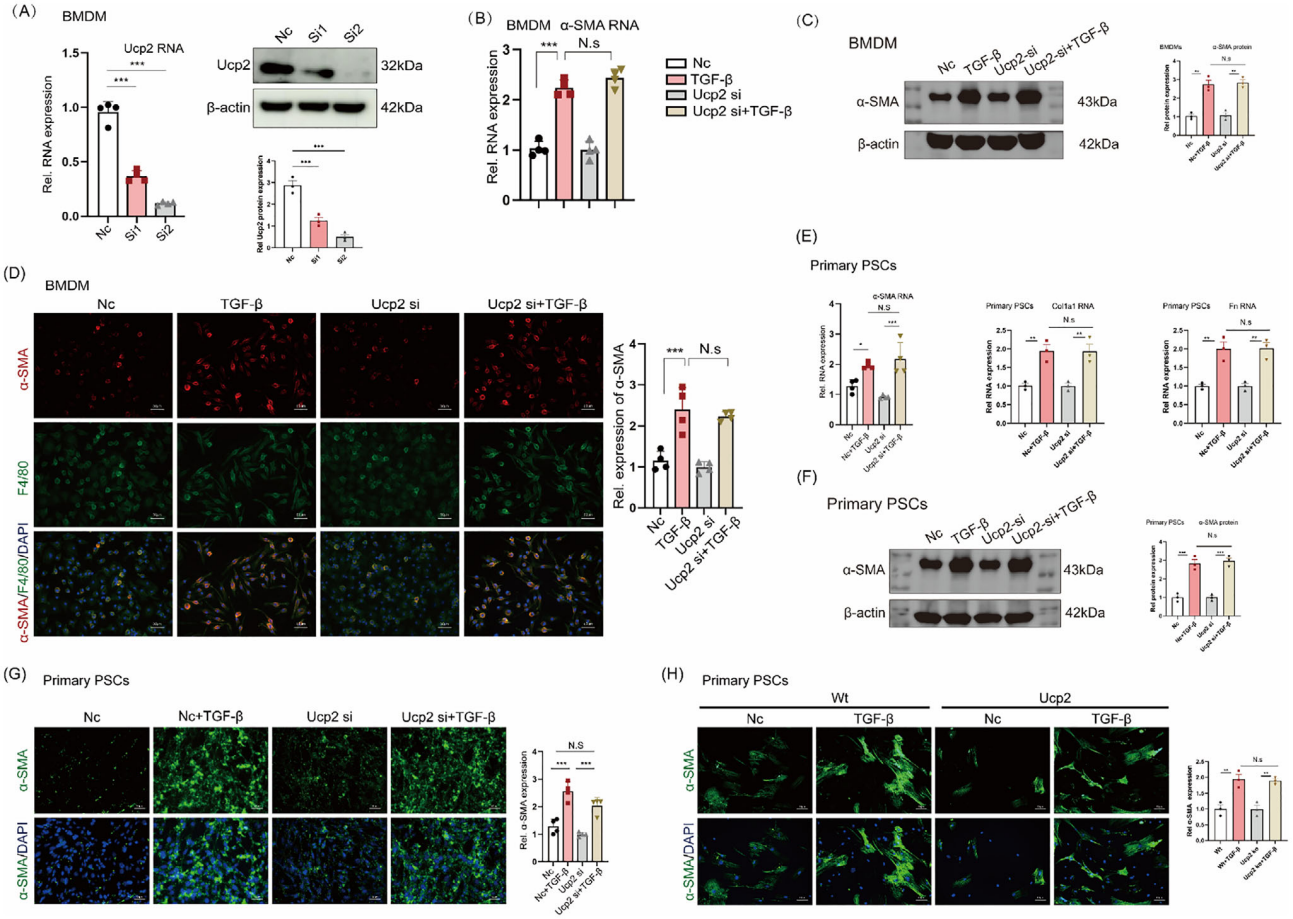
UCP2 knockout does not alter the intrinsic fibrosis‐related phenotype in macrophages or PSCs. A) Validation of two UCP2 siRNAs for effective knockdown in BMDMs. *n* = 4 or 3. B) qPCR shows UCP2 knockout does not affect α‐SMA expression in BMDMs; TGF‐β stimulation increases α‐SMA mRNA levels in both UCP2 KO and WT BMDMs, with no significant differences. *n* = 4. C,D) Western blotting and immunofluorescence confirm similar α‐SMA expression levels in both groups after TGF‐β stimulation. 400x magnification, Scale bar, 50 µm. *n* = 4 or 3. E) qPCR analysis of PSCs shows no effect of UCP2 knockout on PSC sensitivity to TGF‐β, with no significant difference in α‐SMA, Col1a1, and Fn mRNA levels between UCP2‐siRNA and Nc groups after TGF‐β stimulation. *n* = 4. F) Western blot analysis confirmed that UCP2 knockdown does not affect the basal α‐SMA expression or the activation of PSCs. *n* = 4. G) α‐SMA immunofluorescence in primary PSCs from WT mice shows that UCP2 knockdown does not affect PSC activation after TGF‐β stimulation. 400x magnification, Scale bar, 50 µm. *n* = 4. H) α‐SMA staining in PSCs from WT and UCP2 KO mice indicates UCP2 knockout does not alter PSC fibrotic phenotype or response to TGF‐β. 400x magnification, Scale bar, 50 µm. *n* = 4. Data are presented as mean ± SEM of three independent experiments; ^*^ *p* < 0.05, ^**^ *p* < 0.01, and ^***^ *p* < 0.001, NS, no significant difference.

To elucidate how UCP2 knockout inhibits the MMT process in CP, we examined its effects on the fibrosis‐related phenotype of macrophages and PSCs. First, to determine whether UCP2 knockout alters the fibrosis‐related phenotype of macrophages, we developed and validated two UCP2 small interfering RNA (siRNA) molecules for effective UCP2 suppression. The results indicated that SiRNA2 was more effective in knocking down UCP2. Therefore, sequence 2 was used for UCP2 knockdown in subsequent studies (**Figure** **5**A).

We subsequently used TGF‐β to induce the MMT process in BMDMs. The results demonstrated that UCP2 knockdown did not alter the basal expression of α‐SMA in BMDMs or their sensitivity to TGF‐β‐induced MMT. Following TGF‐β stimulation, α‐SMA mRNA levels were significantly elevated in both the UCP2 knockdown and Negative Control (NC) groups, with no significant difference between the groups (Figure 5B). Western blot analysis yielded consistent results (Figure 5C), which were further validated by immunofluorescence analysis (Figure 5D). These data suggest that UCP2 knockdown does not influence the MMT process in macrophages, indicating that UCP2 may indirectly affect the MMT process in CP through interactions with other cell types.

Previous studies have shown that the persistent activation of PSCs is a key driver of CP, prompting us to investigate the impact of UCP2 knockdown on PSCs. qPCR analysis revealed that UCP2 knockdown did not alter the sensitivity of PSCs to TGF‐β, as there were no significant differences in the mRNA expression levels of α‐SMA, Col1a1, and Fn between the UCP2 knockdown and NC groups after TGF‐β stimulation (Figure 5E). Western blot analysis further confirmed that UCP2 knockdown does not affect the basal α‐SMA expression or the activation of PSCs (Figure 5F). Primary PSCs were isolated from WT mice and subjected to siRNA‐mediated UCP2 knockdown. Immunofluorescence analysis of α‐SMA revealed that following TGF‐β stimulation, α‐SMA expression levels remained comparable between PSCs with and without UCP2 knockdown (Figure 5G). Additionally, we further isolated primary PSCs from WT and UCP2 KO mice, and α‐SMA immunofluorescence confirmed that UCP2 knockout did not affect the fibrotic phenotype or the sensitivity of PSCs to TGF‐β stimulation (Figure 5H). Therefore, UCP2 knockout does not impact the intrinsic fibrotic phenotype of macrophages or PSCs. Together with previous findings, UCP2 is primarily localized in pancreatic acinar cells. These results suggest that UCP2 may regulate the MMT process in CP through its role in pancreatic acinar cells.

### 2.5 Metabolomics Analysis Indicates that UCP2 Knockdown Predominantly Influences Lipid Metabolism‐Related Signaling Pathways

**Figure 6:**
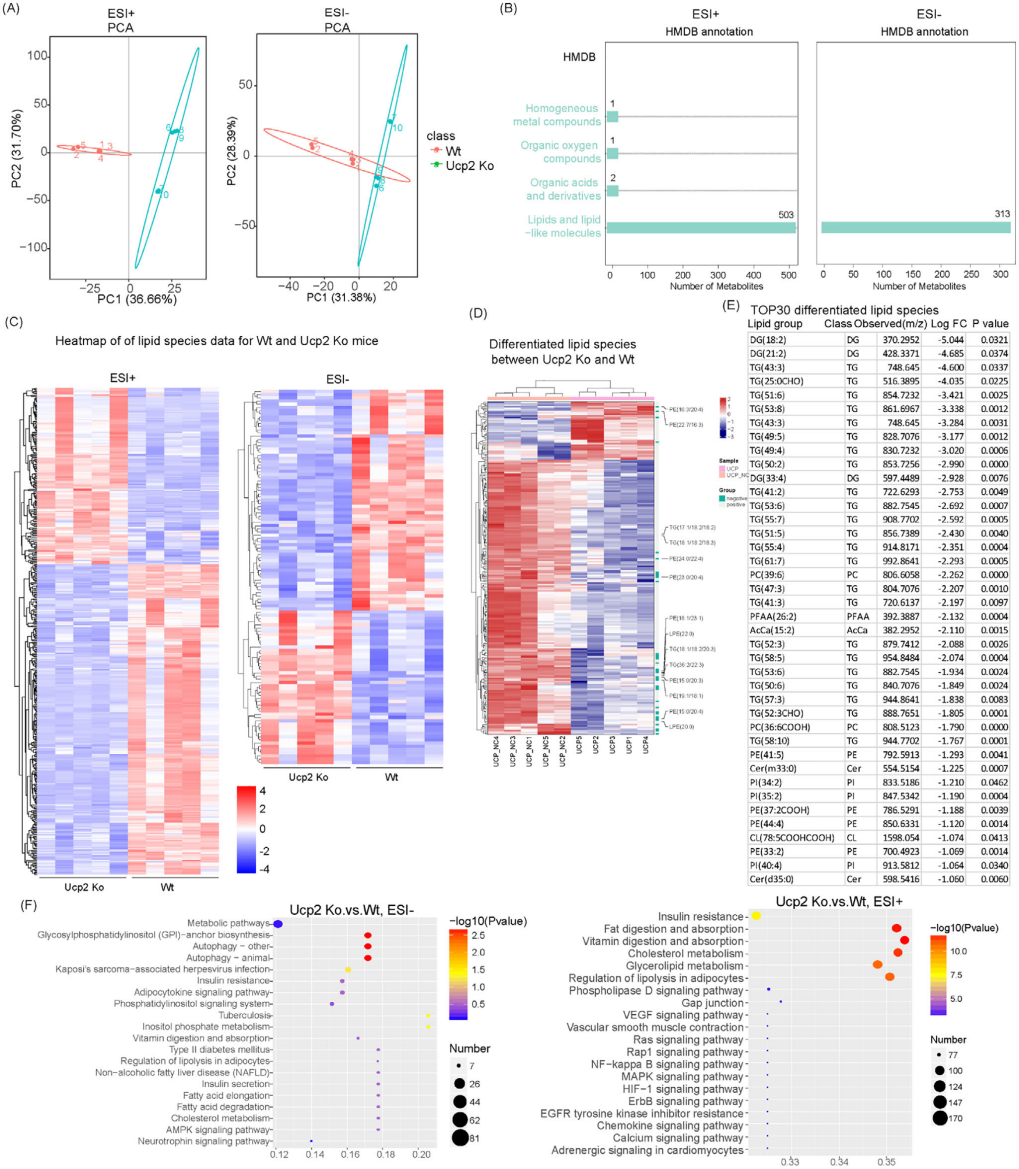
Metabolomics analysis reveals that UCP2 knockdown predominantly affects lipid metabolism‐related signaling pathways. A) PCA effectively distinguishes UCP2 KO mice from WT mice, validating the accuracy of subsequent metabolomic analyses. B) Metabolomic profiling, utilizing the HMDB, identifies significant alterations in lipid and lipid‐like molecules, with 503 and 313 changes observed in ESI+ and ESI‐ modes, respectively, following UCP2 KO. C) A heatmap illustrates the genes with significant differential expression after UCP2 knockout, highlighting key metabolic changes. D) Among the significantly altered lipid species, PE, TG, and LPE exhibit the most pronounced changes. E) The top 30 most significantly altered lipid species are identified, with TG‐related lipids showing the most substantial differences. F) Further enrichment analysis reveals that UCP2 knockout significantly impacts metabolism‐related pathways, particularly those involved in fatty acid digestion and absorption.

To elucidate the regulatory mechanism through which UCP2‐mediated MMT promotes CP progression, we investigated this process from a metabolomics perspective, considering the pivotal role of UCP2 in metabolic regulation.^[^ ^24^ ^]^ We conducted metabolomic sequencing on five pairs of UCP2 knockout and wild‐type mice. Principal component analysis (PCA) effectively distinguished the UCP2 KO group from the WT group, ensuring the accuracy of subsequent analyses (**Figure** **6**A). Using the Human Metabolome Database (HMDB), we identified that UCP2 knockout primarily affects lipid and lipid‐like molecules, with 503 and 313 changes observed in Electrospray Ionization (ESI)^+^ and ESI^−^ modes, respectively (Figure 6B). A heatmap further highlighted the metabolism with significant differential expression following UCP2 knockout (Figure 6C) and the significantly altered lipid species, among which phosphatidylethanolamine (PE), triglycerides (TG), and lysophosphatidylethanolamine (LPE) exhibited the most pronounced changes (Figure 6D). Subsequently, we identified the top 30 most significantly altered lipid species, with TG‐related lipids showing the most notable differences (Figure 6E). Further enrichment analysis revealed that metabolism‐related pathways, particularly those involved in fatty acid digestion and absorption, were significantly altered following UCP2 knockout (Figure 6F). Consequently, in subsequent studies, we focused on elucidating the molecular mechanisms by which UCP2‐mediated MMT contributes to CP progression from the perspective of lipid metabolism.

### 2.6 The Release of LD by Acinar Cells Is Crucial for Initiating the Progression of MMT. UCP2 Knockout Significantly Inhibits Both LD Release and the MMT Process

**Figure 7:**
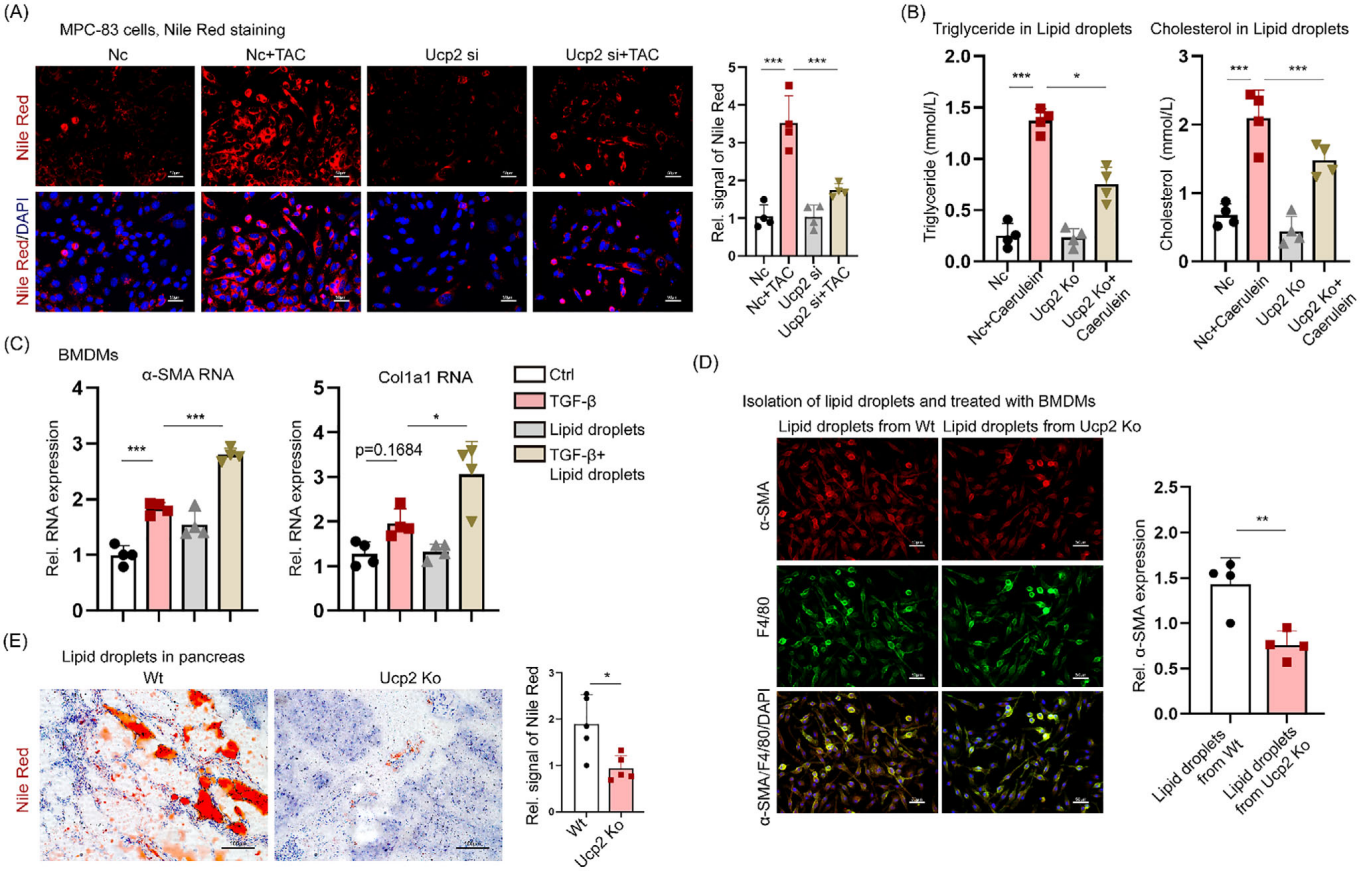
UCP2 knockout inhibits lipid droplet release from acinar cells and the MMT process. A) Nile red staining of MPC‐83 acinar cells stimulated with TAC reveals a significant increase in lipid content, establishing an in vitro model of CP. UCP2 knockout markedly reduces the sensitivity of acinar cells to TAC, counteracting the TAC‐induced increase in lipid levels. 400x magnification, Scale bar, 50 µm. *n* = 4. B) Quantification of TG and cholesterol levels in LD extracted from primary mouse acinar cells shows a significant reduction in lipid content following UCP2 knockout. *n* = 4. C) BMDMs were stimulated with LD isolated from pancreatic acinar cells, TGF‐β alone, or a combination of both. Stimulation with LD or TGF‐β alone led to comparable increases in the mRNA expression levels of α‐SMA and Col1a1. In contrast, co‐stimulation with both LDs and TGF‐β markedly and synergistically enhanced the expression of α‐SMA and Col1a1. *n* = 4. D) LD extracted from WT‐CP and UCP2‐CP mice and co‐cultured with BMDMs reveal that UCP2 knockout significantly inhibits the MMT process in BMDMs. 400x magnification, Scale bar, 50 µm. *n* = 4. E) The lipid content in the pancreas of UCP2 knockout mice is significantly lower than that of WT mice. 400x magnification, Scale bar, 50 µm. *n* = 4. Data are expressed as the mean ± SEM of three independent experiments; ^*^ *p* < 0.05, ^**^ *p* < 0.01, and ^***^ *p* < 0.001.

We investigated how UCP2 knockout inhibits MMT and alleviates CP specifically in acinar cells. Given that UCP2 knockout predominantly influences lipid metabolism‐related signaling pathways, we first stimulated the MPC‐83 cell line with taurocholic acid (TAC) to establish an in vitro CP model. Nile red staining was employed to evaluate lipid content in CP, revealing that TAC markedly increased lipid levels in MPC‐83 cells. In contrast to its effects on macrophages and PSCs, UCP2 knockout significantly diminished the sensitivity of acinar cells to TAC stimulation, thereby counteracting the TAC‐induced increase in lipid levels (**Figure** **7**A). Recent recognition of LD as dynamic organelles crucial for mitigating cellular stress and regulating lipid metabolism has prompted further investigation.^[^ ^8^ ^]^ We extracted LD from primary mouse acinar cells and quantified TG and cholesterol levels. The results demonstrated that UCP2 knockout significantly reduced TG and cholesterol levels in LD from mouse acinar cells (Figure 7B). We further stimulated BMDMs with LD extracted from acinar cells in vitro. Remarkably, LD induced the expression of fibroblast markers α‐SMA and Col1a1 in BMDMs to a degree comparable to TGF‐β stimulation. Additionally, combined stimulation with TGF‐β and LD significantly increased the mRNA levels of α‐SMA and Col1a1 in BMDMs (Figure 7C). Simultaneous stimulation synergistically upregulated the protein expression levels of Fn, Col1a1, and, α‐SMA in BMDMs (Figure S4A, Supporting Information). Notably, when LD was extracted from acinar cells of WT‐CP and UCP2 KO‐CP mice and co‐cultured with BMDMs, UCP2 knockout significantly inhibited the MMT process in BMDMs (Figure 7D). WB analysis also confirmed this result (Figure S4B, Supporting Information). Moreover, the lipid content in the pancreases of UCP2 KO mice was significantly reduced (Figure 7E). In summary, these findings demonstrate that the release of LD by acinar cells is crucial for initiating macrophage MMT, and UCP2 knockout significantly inhibits both LD release from acinar cells and the MMT process in macrophages.

### 2.7 ACSL3 is a Key Regulator of UCP2‐Mediated Lipid Droplet Release in Acinar Cells and MMT Progression

**Figure 8:**
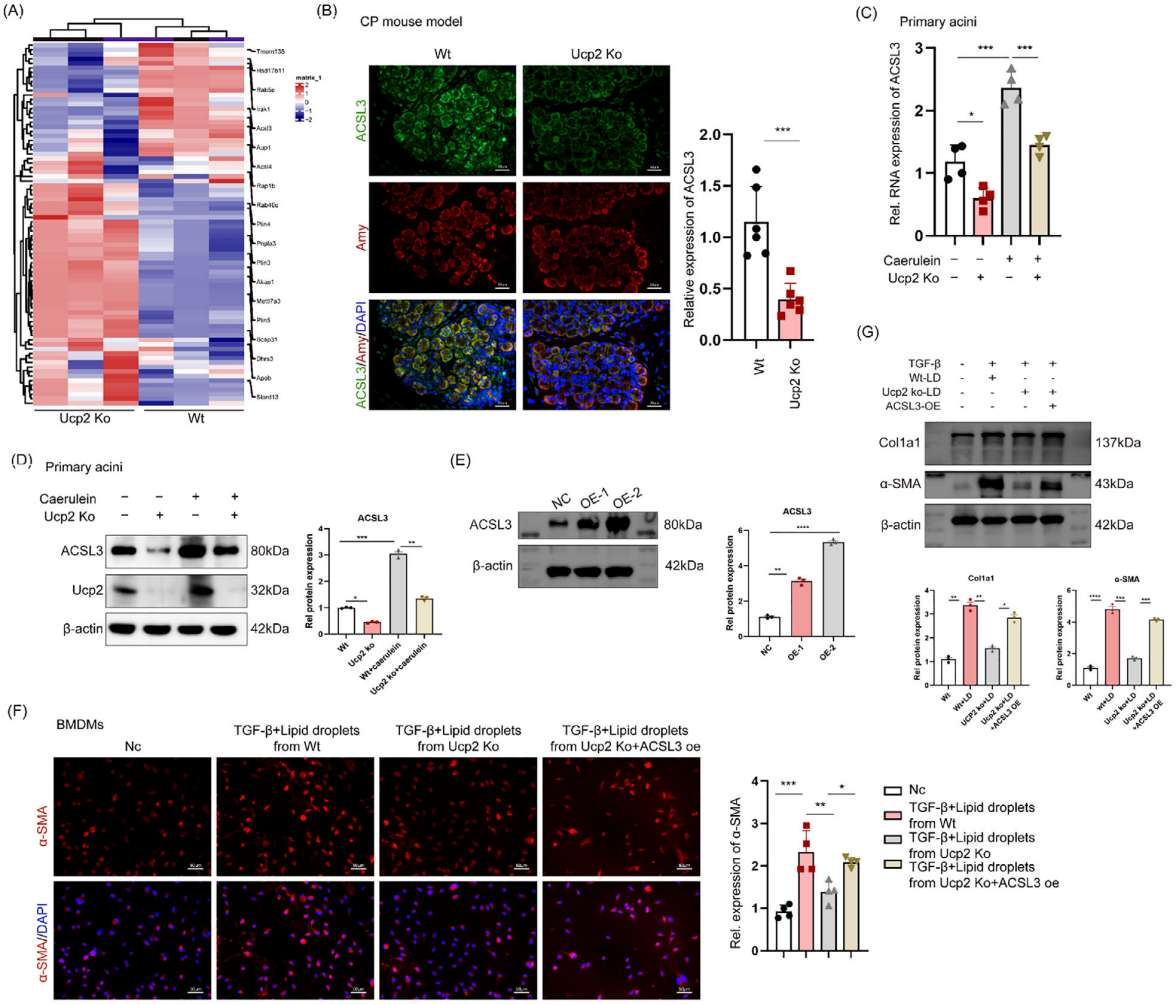
ACSL3 as a key regulator of UCP2‐mediated LD release in acinar cells and MMT Progression. A) Transcriptomic sequencing identifies ACSL3 as a significantly differentially expressed gene in UCP2 knockout acinar cells. B) Immunofluorescence analysis reveals that UCP2 knockout significantly reduces ACSL3 expression in acinar cells within the CP mice model. 400x magnification, Scale bar, 50 µm. n=6. C) qpcR Found Primary acinar cells from UCP2 KO mice show significantly lower baseline ACSL3 expression than WT controls. After caerulein stimulation, ACSL3 expression increases in both groups but remains significantly lower in UCP2 knockout cells. *n* = 4. D) Western blot analysis demonstrates that UCP2 knockout reduces baseline ACSL3 protein expression in primary acinar cells, and although caerulein stimulation upregulates ACSL3 protein levels, this effect is attenuated in UCP2 knockout cells. n=3. E) Successfully overexpressed ACSL3 in primary acinar cells. *n* = 3. F) LD from WT and UCP2 knockout acinar cells stimulated BMDMs with TGF‐β. WT LD increased α‐SMA expression, while UCP2 knockout inhibited it. ACSL3 overexpression reversed this inhibition. 400x magnification, Scale bar, 50 µm. n=4. G) Western blotting revealed that LD from WT and UCP2 knockout acinar cells, when combined with TGF‐β, stimulated BMDMs. WT LD increased α‐SMA expression, while UCP2 knockout inhibited it. ACSL3 overexpression reversed this inhibition. N=3. Data are expressed as the mean ± SEM of three independent experiments; ^*^ *p* < 0.05, ^**^ *p* < 0.01, ^***^ *p* < 0.001, and ^****^ *p* < 0.0001.

To elucidate the mechanism by which UCP2 knockout inhibits lipid droplet release in acinar cells, transcriptomic sequencing identified significantly differentially expressed genes, including Acyl‐CoA Synthetase Long‐Chain Family Member 3 (ACSL3) (**Figure** **8**A). ACSL3 is a key enzyme that catalyzes the esterification of fatty acids with Coenzyme A and plays a crucial role in converting exogenous fatty acids into LD. Its role in lipid metabolism regulation is essential for both the formation and release of LD.^[^ ^25^ ^]^ Therefore, we investigated whether ACSL3 mediates the inhibitory effect of UCP2 knockout on lipid droplet release in acinar cells.

We first assessed ACSL3 expression levels in a CP mouse model, finding that UCP2 knockout significantly suppressed ACSL3 expression in acinar cells (Figure 8B). This finding was further confirmed by immunohistochemical analysis (Figure S5, Supporting Information). We further isolated primary acinar cells from these mice and induced an in vitro CP model using caerulein. Interestingly, qPCR suggested that the baseline expression levels of ACSL3 in UCP2 knockout acinar cells were significantly lower than in those from WT mice. Although caerulein stimulation significantly increased ACSL3 expression in both UCP2 knockout and WT acinar cells, the increase in UCP2 knockout cells remained significantly lower than that observed in WT cells (Figure 8C). We then examined protein expression levels of ACSL3 and UCP2 in primary acinar cells, revealing that UCP2 knockout reduced baseline ACSL3 expression. Although caerulein stimulation upregulated ACSL3 protein expression, this increase was mitigated by UCP2 knockout (Figure 8D).

Thus, ACSL3 may be a critical regulatory factor in UCP2 knockout‐mediated inhibition of LD release in acinar cells. Next, we extracted acinar cells from WT and UCP2 KO mice and overexpressed ACSL3 in primary acinar cells from UCP2 KO mice. We then isolated LD from these cells and stimulated BMDMs with a combination of TGF‐β and LD. We successfully overexpressed ACSL3 in primary acinar cells to investigate its effect on MMT (Figure 8E). LD from WT acinar cells combined with TGF‐β significantly increased α‐SMA expression in BMDMs, while LD from UCP2 knockout cells combined with TGF‐β significantly inhibited α‐SMA expression. Notably, the overexpression of ACSL3 in primary acinar cells reversed the inhibition of α‐SMA expression in BMDMs caused by UCP2 knockout (Figure 8F). Western blot results also showed consistency (Figure 8G). In conclusion, ACSL3 is a key regulator of UCP2‐mediated LD release in acinar cells and MMT progression.

### 2.8 UCP2 Mediates the MMT Progress via the Sirt1/Smad3 Signaling Pathway

**Figure 9:**
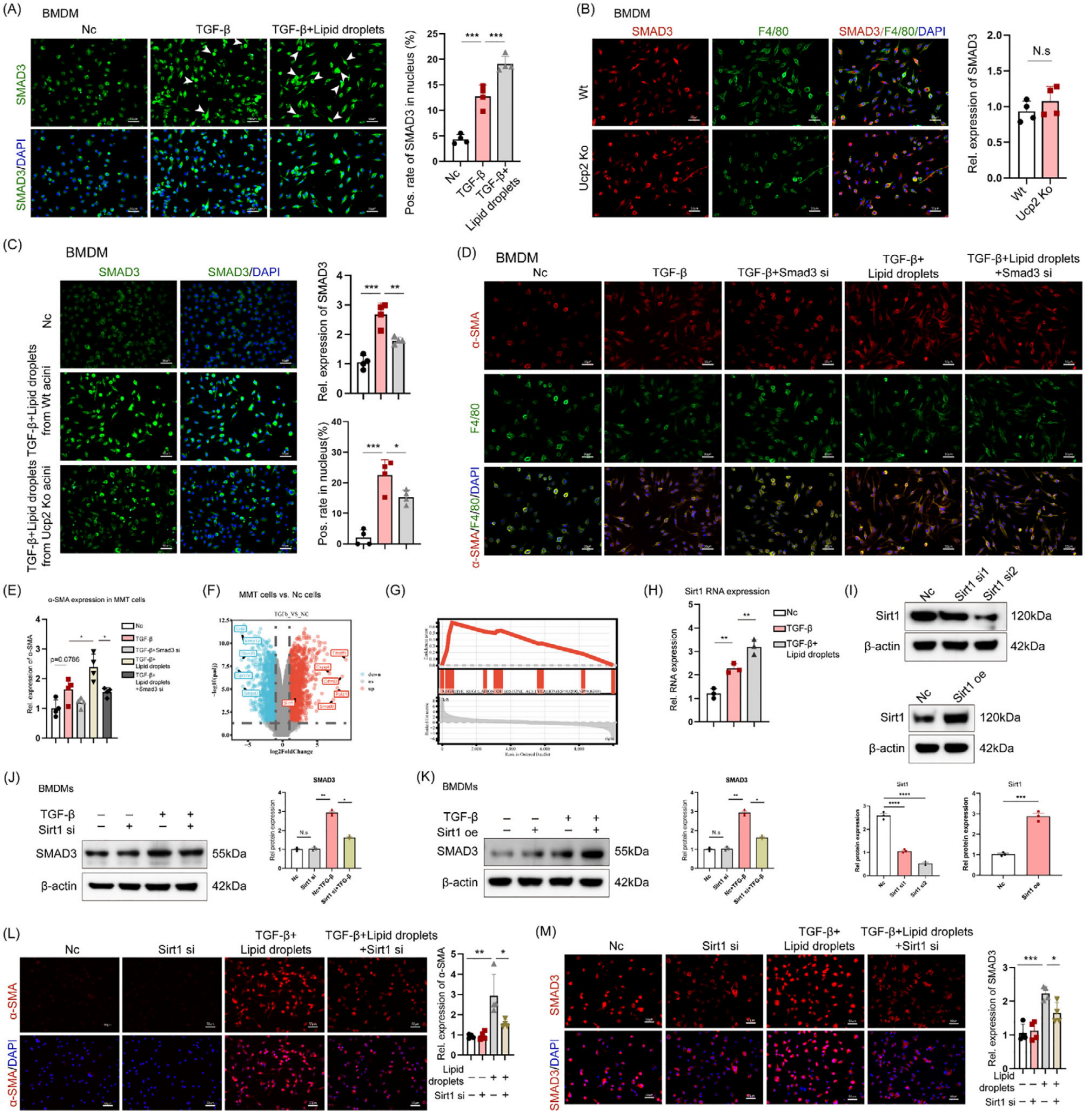
UCP2 mediates the MMT progress via the Sirt1/ Smad3 signaling pathway. A) Immunofluorescence images showing that TGF‐β combined with acinar cell‐derived LD significantly increases Smad3 expression and nuclear translocation in macrophages, compared to TGF‐β alone or in control conditions. 400x magnification, Scale bar, 50 µm. *n* = 4. B) Immunofluorescence demonstrating UCP2 knockout does not affect baseline Smad3 levels in macrophages. 400x magnification, Scale bar, 50 µm. *n* = 4. C) Immunofluorescence demonstrates that UCP2 knockout significantly inhibits the TGF‐β and LD‐induced increase in Smad3 expression in macrophages. 400x magnification, Scale bar, 50 µm. *n* = 4. D,E) Immunofluorescence shows that Smad3 knockdown significantly reduces α‐SMA expression in MMT cells induced by TGF‐β combined with acinar cell‐derived LD, compared to MMT cells induced by TGF‐β alone. 400x magnification, Scale bar, 50 µm. *n* = 4. F) Transcriptomic sequencing of MMT cells and non‐MMT macrophages revealed Sirt1 as a key gene. G) The GSEA analysis suggests that acetylation‐related pathways are significantly enriched after MMT. H) qPCR analysis shows that TGF‐β stimulation significantly upregulates Sirt1 mRNA levels in macrophages, with a more pronounced effect when combined with LD from acinar cells. *n* = 3. I) Successful construction of BMDM cell lines with Sirt1 knockdown and overexpression. J,K) Western blot analysis demonstrates that Sirt1 knockdown counteracts the TGF‐β‐induced upregulation of Smad3 in BMDMs, while Sirt1 overexpression enhances the TGF‐β‐induced increase in Smad3 levels. L) Immunofluorescence results show that Sirt1 knockdown significantly impairs the MMT process by attenuating the TGF‐β and LD‐induced increase in α‐SMA expression in macrophages. 400x magnification, Scale bar, 50 µm. *n* = 4. M) Immunofluorescence results indicate that Sirt1 knockdown reduces the elevation of Smad3 expression induced by LD from acinar cells. 400x magnification, Scale bar, 50 µm. *n* = 4. Data are expressed as the mean ± SEM of three independent experiments; ^*^ *p* < 0.05, ^**^ *p* < 0.01, ^***^ *p* < 0.001, and ^****^ *p* < 0.001 NS, no statistical difference.

Given that LD combined with TGF‐β stimulation synergistically promotes the MMT process, this suggests that UCP2‐mediated MMT progression may not be solely attributed to lipid droplet release from acinar cells. The TGF‐β/Smad3 signaling pathway is the primary regulator of MMT during kidney fibrosis.^[^ ^26^ ^]^ Smad3 is not only a central mediator of TGF‐β signaling in fibrotic diseases, but its deficiency also significantly inhibits the transition of tumor‐associated macrophages into tumor‐associated fibroblasts.^[^ ^27^ ^]^ Targeting Smad3 activity is thus considered a potential antifibrotic therapeutic strategy.^[^ ^28^, ^29^ ^]^ To investigate the regulatory role of Smad3 in the UCP2‐mediated MMT process during CP, we induced the MMT phenotype in BMDMs and assessed Smad3 expression levels. Consistent with previous findings, compared to the control group and TGF‐β stimulation alone, TGF‐β combined with acinar cell‐derived LD significantly increased Smad3 expression and nuclear translocation in macrophages (**Figure** **9**A). Western blot results further confirmed that the combined stimulation of LD and TGF‐β synergistically increased both the total expression and nuclear localization of phosphorylated Smad3 (p‐Smad3) (Figure S6A,B, Supporting Information).

To further explore the impact of UCP2 knockout on Smad3 expression during the TGF‐β and LD‐induced MMT process in macrophages, we first confirmed that UCP2 knockout did not affect baseline Smad3 levels in macrophages (Figure 9B). Western blot analysis also indicated that UCP2 knockdown did not affect the basal expression of Smad3 or p‐smad3 in BMDMs (Figure S6C, Supporting Information). Notably, UCP2 knockout significantly inhibited the TGF‐β and LD‐induced increase in Smad3 expression and nuclear translocation in macrophages (Figure 9C). The Western blot results were consistent with these findings (Figure S6D,E, Supporting Information). To further elucidate Smad3's role in regulating the MMT process, we successfully knocked down Smad3 expression in BMDMs using small interfering RNA and induced the MMT phenotype. The results showed that in MMT cells induced by TGF‐β combined with acinar cell‐derived LD, α‐SMA levels were significantly higher than in MMT cells induced by TGF‐β alone, while Smad3 knockdown significantly reduced α‐SMA expression levels in both conditions (Figure 9D,E). The Western blot results were consistent with these findings (Figure S6F,G, Supporting Information).

Smad3, a well‐established transcription factor, does not directly influence the MMT process. To identify potential upstream and downstream targets of Smad3, we performed transcriptomic sequencing on MMT cells and non‐MMT macrophages, identifying 534 upregulated genes and 387 downregulated genes, including Silent Information Regulator 1 (Sirt1) (Figure 9F). Gene Set Enrichment Analysis (GSEA) further revealed significant differences in pathways related to acetylation modification during MMT (Figure 9G). Acetylation is a crucial regulatory mechanism for LD formation and release, affecting LD regulation by modulating fatty acid metabolism enzymes, LD‐associated proteins, transcription factor activity, energy metabolism balance, and LD degradation. Sirt1, an NAD^+^‐dependent deacetylase, is essential for regulating cellular processes such as metabolism and exerts antifibrotic effects by deacetylating target proteins. We investigated Sirt1 expression in macrophages and found that TGF‐β stimulation significantly upregulated Sirt1 mRNA levels, with a more pronounced effect when combined with LD from acinar cells (Figure 9H). The Western blot results were consistent with these findings (Figure S6H, Supporting Information). We then performed Sirt1 knockdown and overexpression in BMDMs and measured the protein levels of Sirt1 (Figure 9I). TGF‐β stimulation led to significant increases in both Sirt1 and Smad3 expression. While Sirt1 knockdown did not affect Smad3 expression directly, it counteracted the TGF‐β‐induced upregulation of SMAD3 in BMDMs (Figure 9J). Conversely, Sirt1 overexpression did not affect Smad3 expression but significantly enhanced the TGF‐β‐induced increase in Smad3 levels in BMDMs (Figure 9K).

We subsequently evaluated the effects of Sirt1 knockout on the macrophage MMT process and Smad3 expression. Sirt1 knockdown did not affect the basal expression of α‐SMA in macrophages. However, stimulation with LD derived from acinar cells significantly increased α‐SMA expression, an effect substantially attenuated by Sirt1 knockout, indicating that Sirt1 deficiency impairs the MMT process (Figure 9L). Similarly, Sirt1 knockdown did not alter basal Smad3 expression in macrophages, but the elevation of Smad3 expression induced by LD from acinar cells was significantly reduced by Sirt1 knockdown (Figure 9M). The Western blot results were consistent with these findings (Figure S6I, Supporting Information).

To further explore whether Sirt1 knockout inhibits the MMT process and CP fibrosis in vivo, we first generated Sirt1 knockout mice (Sirt1 ko). The results indicated that systemic Sirt1 knockout did not alleviate pancreatic fibrosis (Figure S7A, Supporting Information). Sirt1 is a multifunctional transcription factor involved in the regulation of inflammation, fibrosis, and tumor progression. Increasing evidence suggests that Sirt1 serves as a protective factor in pancreatitis, with Sirt1 agonists shown to suppress both inflammation and fibrosis in this condition.^[^ ^30^, ^31^, ^32^ ^]^ Notably, Sirt1 is significantly upregulated in human CP samples.^[^ ^33^ ^]^ Given the complexity of Sirt1's functions, we hypothesized that systemic knockout might obscure its role in the MMT process. Thus, we created macrophage‐specific Sirt1 knockout mice (Sirt1 cKO). To exclude the potential effects of Sirt1 cKO on macrophages and PSCs, we induced M2 polarization in BMDMs using IL‐4 and co‐cultured them with primary PSCs (Figure S7B, Supporting Information). The results showed that Sirt1 cKO did not alter the fibrotic phenotype of either macrophages or PSCs (Figure S7C, Supporting Information). HE staining revealed that Sirt1 cKO significantly alleviated the fibrotic characteristics of CP and reduced the histological score (Figure S7A, Supporting Information), with consistent findings from Masson staining (Figure S7A, Supporting Information). Interestingly, Sirt1 cKO significantly inhibited the MMT process (Figure S7D, Supporting Information), with further confirmation provided by α‐SMA staining (Figure S7E, Supporting Information). Taken together, these findings suggest that UCP2 modulates the MMT process through the Sirt1/Smad3 signaling pathway.

## 3 Discussion

Results from single‐cell sequencing, fate‐mapping experiments, lineage tracing, and human sample analyses reveal a novel phenomenon, MMT, observed in kidney fibrosis, cardiac fibrosis, vitreoretinal disease, skeletal muscle fibrosis, pulmonary fibrosis, liver fibrosis, and tumor‐associated fibroblast generation.^[^ ^15^, ^16^, ^26^, ^28^, ^34^, ^35^, ^36^, ^37^ ^]^ These findings suggest that MMT may be a universal feature across all fibrosis‐related diseases.^[^ ^14^ ^]^ During pancreatic fibrosis, macrophage polarization has been well characterized,^[^ ^13^ ^]^ yet the phenotype transition of macrophages, particularly the existence and influencing factors of MMT, remains unclear. Furthermore, the interaction between acinar cells and macrophages within the CP fibrotic microenvironment is not fully understood. Our evidence demonstrates that the UCP2 gene upregulates ACSL3 in acinar cells, which promotes LD release and drives the MMT process. Concurrently, UCP2 regulates the Sirt1/Smad3 signaling pathway to induce MMT in macrophages, thereby advancing pancreatic fibrosis (Graphical abstract). Overall, our findings suggest that UCP2 promotes LD formation and release in acinar cells by upregulating ACSL3 expression. This alteration in the local lipid metabolic environment indirectly drives the MMT process. Additionally, UCP2 may regulate the acetylation of Smad3 through Sirt1, enhancing its nuclear activity and activating the TGF‐β/Smad3 signaling pathway. This, in turn, promotes fatty acid metabolism, providing energy for macrophage MMT and synergistically inducing pancreatic fibrosis (Graphical abstract).

Our study is the first to identify MMT in CP. We observed MMT cells in CP mouse models, macrophages, and human CP samples, suggesting that MMT is a novel feature driving CP progression. Indirect evidence for MMT includes the co‐localization of α‐SMA (a marker specific to activated fibroblasts) with macrophage markers (F4/80 or CD68), indicating that macrophages expressing α‐SMA are MMT cells.^[^ ^29^ ^]^ Direct evidence of MMT is provided by lineage tracing and fate mapping experiments, which label and track bone marrow‐derived macrophages.^[^ ^16^ ^]^ Although our study finds that MMT cells constitute only ≈30% of all myofibroblasts—significantly lower than the over 50% reported in renal fibrosis^[^ ^14^, ^16^, ^26^ ^]^—this discrepancy may be due to differing definitions of myofibroblasts. In particular, many atypical cells, such as those derived from bone marrow and endothelial cells, are also classified as myofibroblasts.^[^ ^38^ ^]^ Additionally, the repeated use of caerulein to induce CP may contribute to this difference. Mononuclear macrophages, which exhibit high plasticity, can be categorized into three types: CD11b^+^/Ly6C high macrophages, mainly associated with M1 polarization; CD11b+/Ly6C intermediate macrophages, primarily associated with M2 polarization; and CD11b+/Ly6C low macrophages, which may participate in the MMT process.^[^ ^39^ ^]^ Most MMT cells exhibit characteristics of M2 macrophages, with some uncontrolled M2 macrophages also contributing to MMT.^[^ ^16^ ^]^ Caerulein‐induced CP results from a cumulative effect of acute inflammation. Although fibrosis is a major feature of CP, inflammation remains a significant characteristic in this model, leading to a different macrophage composition compared to renal fibrosis, with a potentially higher proportion of M1 macrophages. Lineage tracing or fate mapping is required to accurately determine the proportion of MMT cells in CP. Notably, blocking MMT significantly inhibits fibrosis in CP, underscoring the crucial role of MMT in mediating CP fibrosis.

Abnormal lipid metabolism, characterized by elevated TG, is a hallmark of CP. This metabolic dysfunction is closely linked to CP risk factors, including chronic alcohol consumption and hyperlipidemia. Moreover, lipid metabolism serves as a key regulator of oxidative stress, contributing to the development of pancreatic fibrosis.^[^ ^7^ ^]^ LD, as organelles for lipid storage, can directly respond to external stimuli and mediate metabolic shifts, including changes in oxidative phosphorylation and aerobic glycolysis, thereby influencing immune cell functions, particularly those of macrophages.^[^ ^40^, ^41^, ^42^ ^]^ The interaction between LD and macrophages is bidirectional. Macrophages can induce LD formation, with the accumulation of these droplets aiding in the phagocytosis of long‐chain fatty acids, thus inhibiting tumor cells' lipid metabolism.^[^ ^43^ ^]^ Conversely, emerging evidence indicates that LD can directly regulate macrophage function, independent of energy alterations resulting from lipid metabolic reprogramming.^[^ ^40^, ^44^ ^]^ Our research demonstrates that LD released by pancreatic acinar cells drives the macrophage MMT process more effectively than TGF‐β stimulation. Furthermore, this release of LD is dependent on the UCP2‐mediated upregulation of ACSL3 expression in acinar cells. Studies have shown that ACSL3 is a crucial driver of exogenous LD formation in renal tumors.^[^ ^25^ ^]^ Moreover, ACSL3 influences both the translocation and release of LD.^[^ ^45^ ^]^ Our research offers new insights into the mechanisms by which acinar cells interact with macrophages to mediate the progression of CP.

As previously discussed, the energy balance between oxidative phosphorylation and aerobic glycolysis is crucial in regulating LD formation, release, and macrophage function. UCP2, a mitochondrial uncoupling protein, is a key regulator in this process. The decoupling of the electron transport chain from phosphorylation during mitochondrial uncoupling is a vital mechanism for regulating reactive oxygen species. In fibrotic environments, unsaturated fatty acids are preferentially utilized as an energy source, and these acids specifically enhance UCP2 activity.^[^ ^46^, ^47^ ^]^ UCP2 is highly expressed in human renal fibrosis samples and mouse models, where it promotes lipid deposition and extracellular matrix formation, contributing to the progression of renal fibrosis.^[^ ^47^ ^]^ By regulating energy metabolism, UCP2 influences macrophage polarization and modulates inflammatory and fibrotic diseases.^[^ ^17^, ^18^, ^19^, ^48^ ^]^ Importantly, our research demonstrates that UCP2's regulation of LD formation and release affects the macrophage MMT process, complementing studies on UCP2's role in lipid metabolism and macrophage function. UCP2 is a gene strongly associated with pancreatic diseases. It controls pancreatic growth and development and regulates the function of pancreatic islet β‐cells and α‐cells.^[^ ^21^, ^46^, ^49^ ^]^ Although UCP2's role in the regulation of pancreatitis has not been directly reported^[^ ^5^, ^50^ ^]^ its influence on metabolic reprogramming in pancreatic cancer is significant.^[^ ^6^ ^]^ Our research is the first to identify UCP2 as a key gene mediating the transition from AP/SAP to CP. While smoking, alcohol consumption, and hyperlipidemia are primary risk factors for CP,^[^ ^1^ ^]^ the critical role of UCP2 in its pathogenesis should not be overlooked, especially given its impact on metabolism and macrophage function. Notably, the UCP2‐regulated MMT process directly contributes to CP fibrosis.

The TGF‐β/ Smad3 signaling pathway plays a pivotal role not only in regulating fibrosis but also in controlling the MMT progress.^[^ ^28^, ^29^ ^]^ Targeting Smad3‐regulated pathways may offer a novel therapeutic strategy for fibrotic diseases.^[^ ^27^ ^]^ Our findings demonstrate that Smad3 upregulation is a critical mediator of the MMT process in chronic pancreatitis CP. As a classical transcription factor, Smad3 is integral to nuclear signal transduction. We observed that LD stimulation from acinar cells markedly increased Smad3 expression and nuclear translocation in macrophages, which correlated with the progression of MMT. Additionally, Smad3 is regulated by Sirt1. Through transcriptome sequencing and GSEA enrichment analysis, we identified Sirt1 as an upstream regulator of Smad3. Sirt1 modulates the progression of various diseases by deacetylating multiple transcription factors. Specifically, Sirt1 deacetylates and inhibits Smad3 activity, thereby preventing the activation of the TGF‐β signaling pathway, reducing collagen production, and limiting extracellular matrix accumulation—mechanisms that have been confirmed in other fibrotic diseases.^[^ ^51^, ^52^ ^]^ Additionally, Sirt1 plays a critical regulatory role in LD metabolism by influencing lipid synthesis, breakdown, and storage.^[^ ^53^ ^]^ Importantly, evidence suggests the presence of a binding site between Sirt1 and the UCP2 promoter. Supporting this, studies in β cells show that Sirt1 binds to the UCP2 promoter and inhibits its transcription, thereby regulating insulin secretion.^[^ ^54^ ^]^ Furthermore, Sirt1 modulates UCP2 function by affecting other promoter binding sites, such as Foxa1 and PPARγ.^[^ ^55^ ^]^

In summary, our research identifies UCP2 as a susceptibility gene for CP, particularly in cases evolving from AP or SAP. The MMT progress is a newly recognized feature of CP, and its inhibition markedly alleviates CP progression. Our findings suggest that UCP2 regulates the MMT process by upregulating ACSL3 in pancreatic acinar cells, which promotes LD release that drives macrophage MMT, and by enhancing the Sirt1/ Smad3 signaling pathway to further support MMT. Targeting the UCP2‐driven MMT process could offer a novel therapeutic strategy for CP.

## 4 Experimental Section

### Mouse Pancreatitis Models

All experimental procedures were approved by the Animal Experiment Ethics Committee of Renmin Hospital of Wuhan University (Ethics Approval No. WDRM (Fu) 20240405D). WT mice were purchased from Wuhan Slyke Lab Animal Co., Ltd., while UCP2 knockout (UCP2‐/‐) mice were generated by Cyagen Biosciences, Suzhou. The UCP2‐/‐ mice were subsequently bred, genotyped, and utilized in experiments at Renmin Hospital of Wuhan University. In agarose gel electrophoresis, UCP2 homozygous mice showed only a single band at 493 bp; heterozygous mice displayed three bands at 493 bp, 465 bp, and 1855 bp; while wild‐type mice exhibited two bands at 465 bp and 1855 bp. The AP model was established using previously reported methods.^[^ ^56^ ^]^ The SAP model was created by administering a single intraperitoneal injection of 10 mg kg^−1^ body weight LPS per mouse, in addition to the AP protocol. The CP model was developed according to the procedures outlined in the literature. For detailed information, please refer to the supplementary materials.

### Human Pancreas Samples

Four human chronic pancreatitis samples and four normal pancreatic samples were collected from Renmin Hospital of Wuhan University. All samples were used following the guidelines approved by the Ethics Committee of Renmin Hospital of Wuhan University (Ethics Approval Number: WDRY‐2024‐K115).

### Histological Analysis

Mouse pancreatic samples were fixed in paraformaldehyde after collection, embedded in paraffin, and then sectioned. Human samples were directly sectioned from wax blocks. Subsequently, HE staining and Masson's trichrome staining were performed. The HE staining score was assessed according to the previously published article.^[^ ^56^ ^]^

### Pancreatic Enzyme Activity Measurement

Mouse blood samples were first anticoagulated and then centrifuged to obtain the supernatant. The supernatant was subsequently incubated with reagents according to the instructions provided in Siemens' amylase and lipase assay kits. The absorbance was measured using a microplate reader, and enzyme activity units were calculated based on the standard curve and the absorbance values.

### Cell Isolation and Culture

Primary acinar cells and bone marrow‐derived macrophages were isolated from WT and UCP2 knockout mice following the previously established methods.^[^ ^56^ ^]^ PSCs were extracted using standard protocols.^[^ ^57^ ^]^ All cells were cultured according to their specific growth requirements, with detailed procedures available in the supplementary materials. TGF‐β (10 ng mL^−1^) was used to stimulate BMDMs and acinar cells. MPC‐83 cells, purchased from Shanghai Yaji Biotechnology Co., Ltd., were cultured in 1640 medium according to standard cell culture procedures.

### Multicolor Immunofluorescence

Tissue samples from humans and animals were sectioned and subsequently stained. For cellular samples, staining was performed after the cells had adhered to slides. The detailed procedures follow those described in previous literature. In this study, F4/80 was utilized to label mouse macrophages, CD68 to label human macrophages, α‐SMA to label activated fibroblasts, Amy to label acinar cells, Krt19 to label ductal cells, and DAPI to label cell nuclei. Additionally, immunofluorescence was employed to assess the expression of Smad3 and Col1a1. All antibodies were procured from Abcam. Fluorescence images were acquired using a fluorescence microscope and processed with Photoshop. Relative fluorescence intensities were independently assessed and compiled by two researchers.

### RNA and Protein Extraction, qPCR, and Western Blot (WB) Experiments

RNA was extracted from cells and tissues using standard protocols. After verifying RNA quality, it was reverse‐transcribed into cDNA. qPCR was performed with β‐actin as the internal control, using primers for β‐actin(5′‐TGTTACCAACTGGGACGACA‐3′and 5′‐GGGGTGTTGAAGGTCTCAAA‐3′), α‐SMA(5′GTGCGGACAGGAATTGAAGC‐3′ and 5′GTTCCGCTCCTCTCTCCAAC‐3′), Col1a1(5′‐CTCAAGGTCACGGTCACGAA‐3′ and 5′TTCTCCTGGCAAAGACGGAC‐3′), UCP2(5′‐TGTGGTTCGATGGGAGGCAC‐3′ and 5′‐TAAGTGTTTCGTCTCCCAGCCAT‐3′), and sirt1(5′‐ACAATCTGCCACAGCGTCAT‐3′ and 5′‐TCGGCTACCGAGGTCCATA‐3′) For protein extraction, standard procedures were followed, with β‐actin as the internal control. SDS‐PAGE was conducted with a 10% gel, followed by electrophoresis, membrane transfer, blocking, and overnight incubation with the primary antibody. The next day, the membrane was incubated with the secondary antibody and developed. Band intensity was analyzed using ImageJ software. Primary antibodies for UCP2, Sirt1, Col1a1, Fn, Smad3, p‐Smad3, β‐actin, PCNA, and ACSL3 were obtained from Wuhan Protein Tech Biotechnology Co., Ltd.

### SiRNA and Adenovirus Transfection

siRNAs targeting UCP2 and Sirt1, along with adenoviruses for ACSL3 overexpression, were obtained from Shanghai Miaoling. UCP2 was knocked down in BMDMs, while Sirt1 was knocked down, and ACSL3 was overexpressed in primary acinar cells. The effectiveness of these genetic modifications was subsequently verified.

### Metabolomics and Transcriptomics Sequencing—Metabolomics Analysis

Metabolomics analysis was performed using liquid chromatography‐mass spectrometry (LC‐MS). Samples were homogenized in an ice bath, centrifuged, and the supernatant was evaporated to dryness. The residues were then reconstituted in a 50% methanol solution before LC‐MS analysis. Chromatographic separation and mass spectrometry were carried out, with data acquired in both positive and negative ionization modes. Metabolites were identified and quantified, with results compared to a standard library. Transcriptomics Analysis: Transcriptomic analysis was conducted using RNA‐Seq. After RNA extraction, the quality and quantity of RNA were assessed. Library preparation and sequencing were performed using 150 bp paired‐end reads. Following adapter removal and filtering of low‐quality sequences, clean reads were aligned and quantified against the reference genome, followed by differential expression analysis. Metabolomics and transcriptomics data were analyzed using R software. Data were normalized, and PCA and hierarchical clustering were used to identify significant patterns and associations. Additionally, pathway enrichment analysis was performed to elucidate the biological significance of the observed changes.

### Isolation and Culture of LD from Primary Pancreatic Acinar Cell Supernatants

Primary pancreatic acinar cells were isolated from mice using methods described in the previously published work.^[^ ^56^ ^]^ Once the acinar cells adhered and reached an appropriate confluence, the culture medium was collected, and the supernatant was subjected to differential centrifugation to separate LD. The lipid‐rich particles were then resuspended in a buffer solution and transferred to a lipid‐free medium to prevent contamination and degradation. Essential nutrients and growth factors were added to the medium as required. The morphology and quantity of the LD were regularly assessed using microscopy and lipid quantification assays.

### Nile Red Staining Method

Acinar cells were fixed with 4% paraformaldehyde at room temperature and washed thoroughly with PBS. The cells were then stained with a 1 µg mL^−1^ Nile Red solution and incubated at 37 °C for 30 min to ensure adequate staining. After incubation, the cells were washed again with PBS. The stained cells were then observed and photographed under a microscope.

### Statistical Methods

A detailed description of the statistical methods is provided in the supplementary materials.

## Conflict of Interest

The authors declare no conflict of interest.

## Author Contributions

K.W., L.Z., and B.D. contributed equally to this work. K.W. and W.W. conceived the study. K.W., L.Z., B.D., W.J., and C.C. were responsible for patient enrollment and sample collection. Q.S., K.W., K.Z., L.Z., T.K., J.H., and B.D. performed experiments and data analysis. W.J., J.H., and C.C. conducted independent statistical analyses and data visualization. K.W. and W.W. drafted the original manuscript. J.H., B.D., L.Z., K.Z., and Q.S. contributed to the review and revision of the manuscript. J.H. led the revision process, refining the experimental design and enhancing the overall manuscript structure. All authors reviewed and approved the final version of the manuscript.

## Supporting information



Supporting Information

## Data Availability

The data that support the findings of this study are available from the corresponding author upon reasonable request.
