# Supplementary material for: UCP2 Upregulates ACSL3 to Enhance Lipid Droplet Release from Acinar Cells and Modulates the Sirt1/Smad3 Pathway to Promote Macrophage‐to‐Myofibroblast Transition in Chronic Pancreatitis
Source: Adv Sci (Weinh). 2025 Aug 28;12(43):e12571. doi: 10.1002/advs.202412571 (PMC12631888; doi:10.1002/advs.202412571)
Supplement: Supplementary file 1 — Supporting Information [file ADVS-12-e12571-s001.docx]

Supporting Information

*UCP2 Upregulates ACSL3 to Enhance Lipid Droplet Release from Acinar Cells and Modulates the SIRT1/SMAD3 Pathway to Promote Macrophage-to-Myofibroblast Transition in Chronic Pancreatitis*

Kunpeng Wang^1,2,3 #^, Lilong Zhang^1,2,3 #^, Beiying Deng^3,4#^, Wanrong Jiang^1,2,3^, Tianrui Kuang^1,2,3,^ Chen Chen^1,2,3^, Kailiang Zhao ^1,2,3*^, Qiao Shi ^1,2,3*^, Jun He^5*^, Weixing Wang^1,2,3**^

This file includes:

Supplementary Experimental Section

Figure S1. Comparison of Amy, Insulin, and Krt19 immunofluorescence staining in UCP2 KO and WT mice at 1-6 weeks of age.

Figure S2. Comparison of HE staining, pancreatic endocrine and exocrine function, and the expression of pancreatic extracellular matrix in UCP2 KO and WT mice at 1-6 weeks of age.

Figure S3. Multiplex immunofluorescence results of MMT cells.

Figure S4. Supplementary WB results related to LD.

Figure S5. Supplementary Immunohistochemistry results are related to Figure 8B.

Figure S6. Supplementary WB results are related to Figure 9.

Figure S7. Macrophage-specific knockout of Sirt1 inhibits MMT and pancreatic fibrosis.

Supplementary Experimental Section

***Induction of pancreatitis*:**

**Acute pancreatitis (AP) model** (**Figure 1A**): Mice underwent twelve intraperitoneal injections of caerulein (100 μg/kg; Shanghai Yuanye CAS: 31362-50-2), each administered at one-hour intervals, following 12 hours of fasting, with free access to water. Pancreatitis phenotypes peaked 12 hours after the first caerulein injection, at which point mice were humanely euthanized^1^.

**Severe acute pancreatitis (SAP) model** (**Figure 1A**): Mice underwent twelve intraperitoneal injections of caerulein (100 μg/kg; Shanghai Yuanye CAS: 31362-50-2), each administered at one-hour intervals, following 12 hours of fasting, with free access to water. After the final intraperitoneal injection of frogspin, an additional intraperitoneal injection of LPS (Sigma EC:297-473-0) at 10 mg/kg body weight was administered to the mouse. Pancreatitis phenotypes peaked 12 hours after the first caerulein injection, at which point mice were humanely euthanized^1^

**Chronic pancreatitis (CP) model** (**Figure 1A**): The CP model involved six daily intraperitoneal injections of caerulein (50 μg/kg/hr), administered three days per week for four consecutive weeks. Mice were sacrificed seven days after the last injection.

Blood samples for measuring serum trypsin and amylase activity were obtained from the ophthalmic venous plexus. Pancreatic tissues were harvested and weighted to determine the pancreas/body weight ratio (mg/g), indicative of pancreatic edema or atrophy.

The histological scoring and fibrosis scoring of pancreatic tissue were based on these two References^2,3^.

***Acinar cell preparation:***

The protocol for the isolation of acinar cells from mice was modified from a previous report. Briefly, mice were killed by CO_2_ asphyxiation, and the complete pancreas was collected. Collagenase IV solution (HBSS 1× containing 10 mM HEPES, 200 U/ml collagenase IV, and 0.25 mg/ml trypsin inhibitor) was added to digest the pancreas for 40 min on a shaker (80 rpm/min). Then, stop solution (HBSS 1× containing 5% FBS and 10 mM HEPES) was added to stop the enzymatic reaction. The sample was centrifuged for 3 min at 250 × g, resuspended with 10 ml of complete medium (Waymouth’s medium containing 2.5% FBS, 1% pen/strep, 0.25 mg/ml of trypsin inhibitor, and 25 ng/ml of recombinant human epidermal growth factor), passed through a 100-μm filter, and collected the isolated acini. The acini were cultured with a complete medium at 37 °C under a 5% (v/v) CO_2_ atmosphere.

***BMDMs preparation:***

The femur and tibia of C57BL/6J mice and UCP2 ^KO^ mice were cut under sterile conditions. BM was washed out with sterile DMEM three times. The medium was passed through a cell 70-μm strainer, washed two times with sterile PBS, counted, and seeded in six-well plates or chamber slides for immunofluorescence staining with DMEM containing 10% FBS, 1% pen/strep, and 20 Ng/ml MCSF. Six hours later, the nonattached cells were removed. Cells were ready to use after a culture of 7 days.

***MMT cells induce:***

Bone marrow cells were isolated from the femurs and tibias of UCP2 WT or KO C57BL/6J mice by flushing with Dulbecco's modified Eagle medium (DMEM)/F12 medium. The following day, the cell suspension was transferred into new culture flasks to avoid fibroblast contamination and then differentiated into macrophages by a 5-day stimulation in DMEM/F12 medium containing 10% fetal bovine serum (FBS) and 10 ng/mL macrophage colony-stimulating factor. Subsequently, MMT was induced in second-generation BMDMs by stimulating with 5 ng/mL TGF-β1 in DMEM/F12 containing 1% FBS. Five days after TGF-β1 stimulation, MMT-derived myofibroblasts were identified by immunofluorescence based on coexpression of macrophage (F4/80 or CD68) and myofibroblast (α-SMA) markers.

***Macrophage Depletion Experiment:***

Clodronate, encapsulated in liposomes, is utilized as a macrophage depletion agent. Upon uptake by macrophages, the liposomes release clodronate, which subsequently induces cell death. To control for potential interference from the liposomes, a liposome-only control group is included. Both the macrophage depletion solution and the control solution are stored in a barrier-clean room and thoroughly mixed by inverting the vials more than ten times before injection. Mice are intraperitoneally injected with 0.2 mL of the solution every two days until the completion of the CP model.

***Isolation of Splenic Macrophages and Flow Cytometry Analysis:***

**Cell Isolation:** After isoflurane anesthesia, the spleen is excised and transported in an ice-cold preservation solution. The spleen is then ground to remove red blood cell clumps, and 10 mL of Cell Staining Buffer is used to wash the tissue, with the filtrate collected in a centrifuge tube. Centrifuge at 300g for 5 minutes and discard the supernatant. Add 2 mL of ACK lysing buffer to lyse red blood cells, incubate at room temperature for 2-3 minutes, then add 10 mL of buffer to stop the reaction. Centrifuge again at 300g for 5 minutes and discard the supernatant. Resuspend the pellet in 5 mL of buffer, filter, count the cells, and adjust the concentration to 1×10⁷ cells/mL.

**Staining Procedure:** Take 100 μL (~1×10⁶ cells) of the cell suspension and place it in a 2 mL tube → Add 1 μg of CD16/32 blocking reagent and incubate at room temperature in the dark for 10 minutes → Add surface fluorescent antibodies and incubate at 4°C in the dark for 30 minutes → Wash with 2 mL of buffer, centrifuge at 300g for 5 minutes, and discard the supernatant → Resuspend the pellet in 0.2 mL PBS, add DAPI (excluding the blank control), incubate at 4°C in the dark for 5 minutes, filter, and perform flow cytometry analysis.

**Data Analysis:** Use FSC-H vs SSC-H to define the monocyte/macrophage population (high SSC, moderate FSC) → Exclude aggregated cells using FSC-H vs FSC-A → Exclude dead cells using DAPI staining → F4/80-APC and CD11b-FITC double-positive cells are identified as splenic macrophages.

**The list of primers for matrix-related markers:**

| **Gene** | F | R |
| --- | --- | --- |
| m-α-SMA | CTGACAGAGGCACCACTGAA | CATCTCCAGAGTCCAGCACA |
| m-Col1a1 | AAGAGGCGAGAGAGGTTTCC | AGAACCATCAGCACCTTTGG |
| m-UCP2 | TGTGGTTCGATGGGAGGCAC | TAAGTGTTTCGTCTCCCAGCCAT |
| m-Fn-1 | CTTGCACGATGATATGGAGA | AGCTGAACACTGGGTGCTAT |
| m-TGF-β | CATGGAGCTGGTGAAACGGA | GGCGAGCCTTAGTTTGGACA |
| m-MMP2 | TTTCTATGGCTGCCCCAAGG | GTCAAGGTCACCTGTCTGGG |
| m-Timp1 | ACGAGACCACCTTATACCAG | GCTTTCCATGACTGGGGTGT |
| m-β-actin | TGTTACCAACTGGGACGACA | GGGGTGTTGAAGGTCTCAAA |

Sirt1 knockdown sequence 1: 5'-TACTTCTGTTGAGCAACGTCTCA-3'

Sirt1 knockdown sequence 2: 5'-TACTTTTGTTCAGCAACATCTCA-3'

Smad3 knockdown sequence: 5'-CTGTGAGTTTGCCTTCAACATGA-3'

**Separation of Nuclear and Cytoplasmic Proteins and Western Blot (WB) Experiment**

Cells are cultured to an appropriate density and washed twice with cold PBS to remove culture medium components. Lysis buffer containing protease inhibitors is added to fully suspend the cells, which are then incubated on ice for 15 minutes to lyse the cell membrane. The cell lysate is centrifuged at 800 × g for 5 minutes at 4°C, and the supernatant (cytoplasmic protein fraction) is collected into a new EP tube and stored at -80°C for later use.

The remaining pellet, containing the nuclear fraction, is resuspended with nuclear extraction buffer and vortexed for 15 minutes to facilitate nuclear membrane lysis. After centrifugation at 12,000 × g for 10 minutes at 4°C, the supernatant is collected as the nuclear protein fraction. Protein concentrations of both nuclear and cytoplasmic fractions are measured using the BCA assay and adjusted to the same concentration. An appropriate amount of 5× SDS-PAGE loading buffer is added to the protein samples, which are boiled for 5 minutes to denature and then stored at -80°C.

Western blot is performed to detect the nuclear protein marker PCNA and the cytoplasmic protein marker β-actin, confirming the purity of the two fractions. The main steps of the WB experiment follow the protocol described previously.

**Construction and Breeding of Sirt1 Conditional Knockout (cKO) Mice:**

The wild-type (WT) mice used in this study were purchased from Jiangsu Jicui Yaokang Company. Sirt1 whole-kidney knockout mice (Sirt1 KO) and macrophage-specific Sirt1 knockout mice (Sirt1 cKO) were purchased from Suzhou Saiye Co., Ltd. All animals were housed in SPF-grade animal facilities at Rooms 2 and 4, Building 1, Animal Facility of the First Clinical College, Wuhan University. The animal experiments were approved by the Ethics Committee of Wuhan University, with the ethical approval number WDRM-Animal (Fu) No. 20240405D. The approval is valid from April 10, 2024, to April 19, 2025.

Using the Cre-loxP recombination system, macrophage-specific Sirt1 knockout mice (Sirt1^flox/flox; Lyz2-Cre) were generated. Homozygous Sirt1^flox/flox mice (Sirt1^fl/fl) were crossed with Lyz2-Cre transgenic mice to obtain Sirt1^fl/fl;Lyz2-Cre mice (Sirt1^MΦ-KO). Sirt1^fl/fl mice served as the control group. Genotyping of mice was performed by PCR, and the knockout efficiency of Sirt1 in bone marrow-derived macrophages (BMDMs) was verified by qPCR and Western blot.

**Cell Isolation and Experiment:**

BMDMs were isolated from 6–8-week-old Sirt1^MΦ-KO and control mice. BMDMs were cultured for 7 days in DMEM supplemented with 10% FBS and 20 ng/mL M-CSF to induce maturation. The knockout efficiency of Sirt1 in macrophages was confirmed by qPCR and Western blot.

***Statistical Analysis:***

Data were analyzed using statistical software such as R, SPSS, or GraphPad Prism. For comparisons between groups, parametric tests, including Student's t-test or ANOVA, were employed for normally distributed data, while nonparametric tests, such as the Mann-Whitney U test or Kruskal-Wallis test, were used for non-normally distributed data. Post hoc analyses following ANOVA or Kruskal-Wallis tests were conducted using Tukey's or Dunn's tests, respectively. Correlations between variables were evaluated using Pearson's or Spearman's correlation coefficients, based on data distribution. Statistical significance was set at a p-value less than 0.05, and all tests were two-tailed unless otherwise specified.

Figure S1 Comparison of Amy, Insulin, and Krt19 immunofluorescence staining in UCP2 KO and WT mice at 1-6 weeks of age.


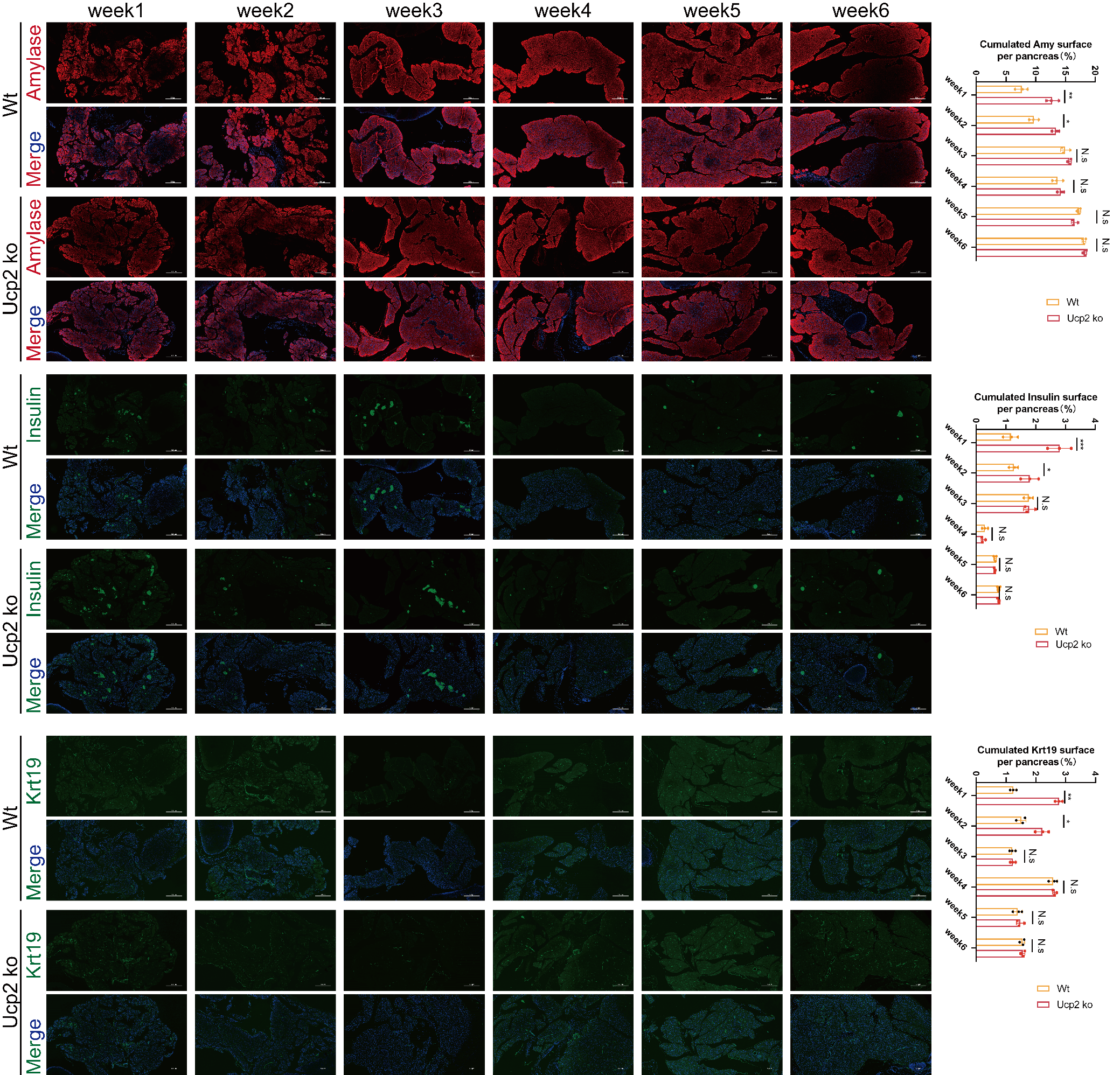
 To further explore the effect of UCP2 gene knockout on pancreatic development, we employed Amy to label acinar cells, Insulin to label β-cells, and Krt19 to label duct cells. Various parameters were compared between age-matched mice (1–6 weeks). Immunofluorescence analysis revealed that at 1 and 2 weeks of age, UCP2 KO mice exhibited significantly larger absolute surface areas of Amy-, Insulin-, and Krt9-positive cells compared to WT mice. However, from 3 weeks onward, no significant differences were observed between the two groups. 40X magnification, scale bar, 500 μm or 50 μm. n=6.Data are expressed as the mean ± SEM of three independent experiments. **P* < 0.05, ** *P* < 0.01, NS, no significant difference.n=3.

Figure S2. Comparison of HE staining, pancreatic endocrine and exocrine function, and the expression of pancreatic extracellular matrix in UCP2 KO and WT mice at 1-6 weeks of age.


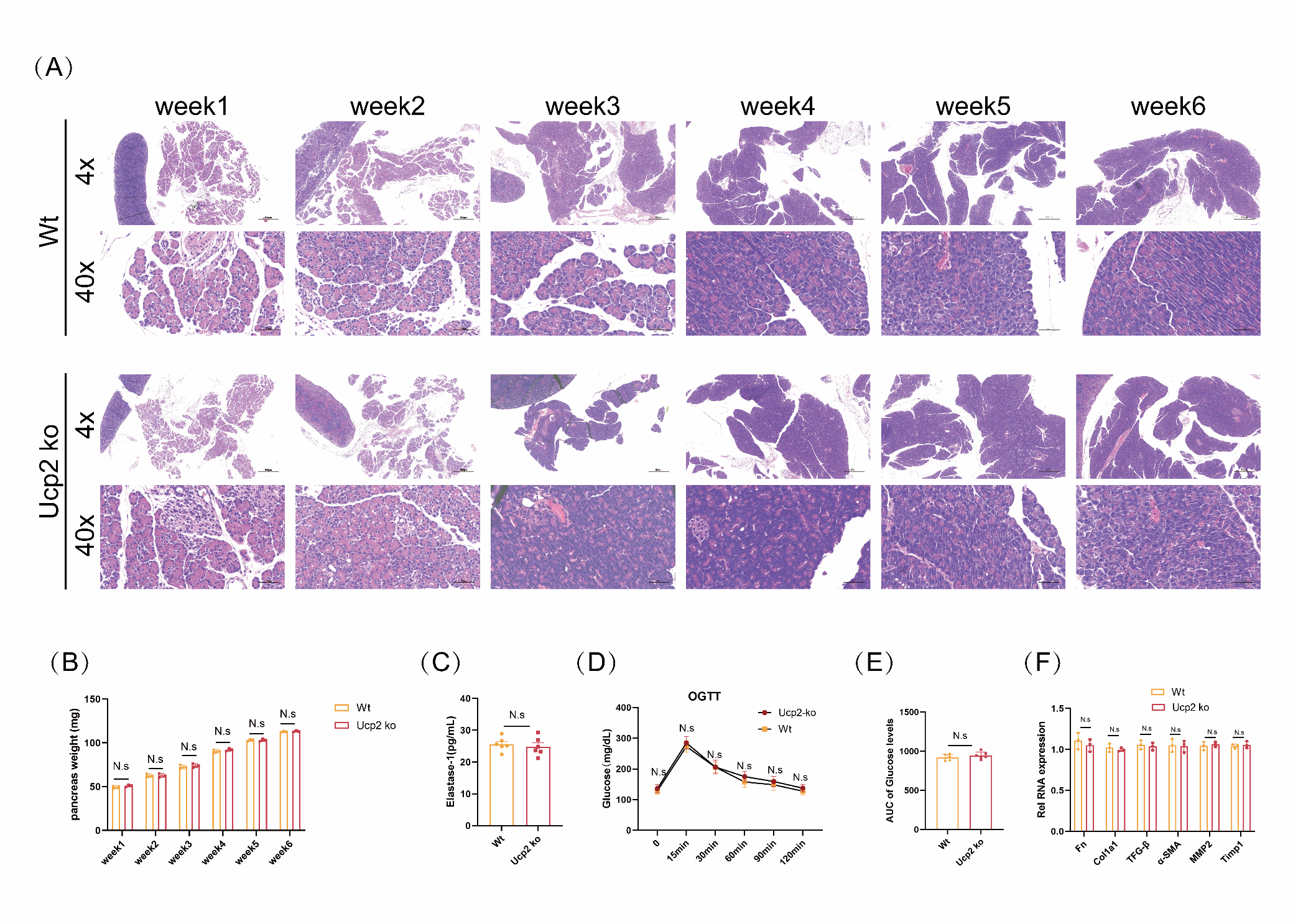


(A)HE staining conducted during weeks 1–6 showed no significant differences in the morphology or structure of the pancreas between age-matched UCP2 KO and WT mice. 40X or 400X magnification, scale bar, 500 μm or 50 μm. n=6. (B) pancreatic weight did not vary significantly between the two groups.n=6. (C-F) By week 6, no differences were observed in pancreatic FE-1 levels, OGTT results, AUC, or extracellular matrix marker expression. n=3. Data are expressed as the mean ± SEM in three independent experiments. NS, no significant difference.

Figure S3. Multiplex immunofluorescence results of MMT cells.


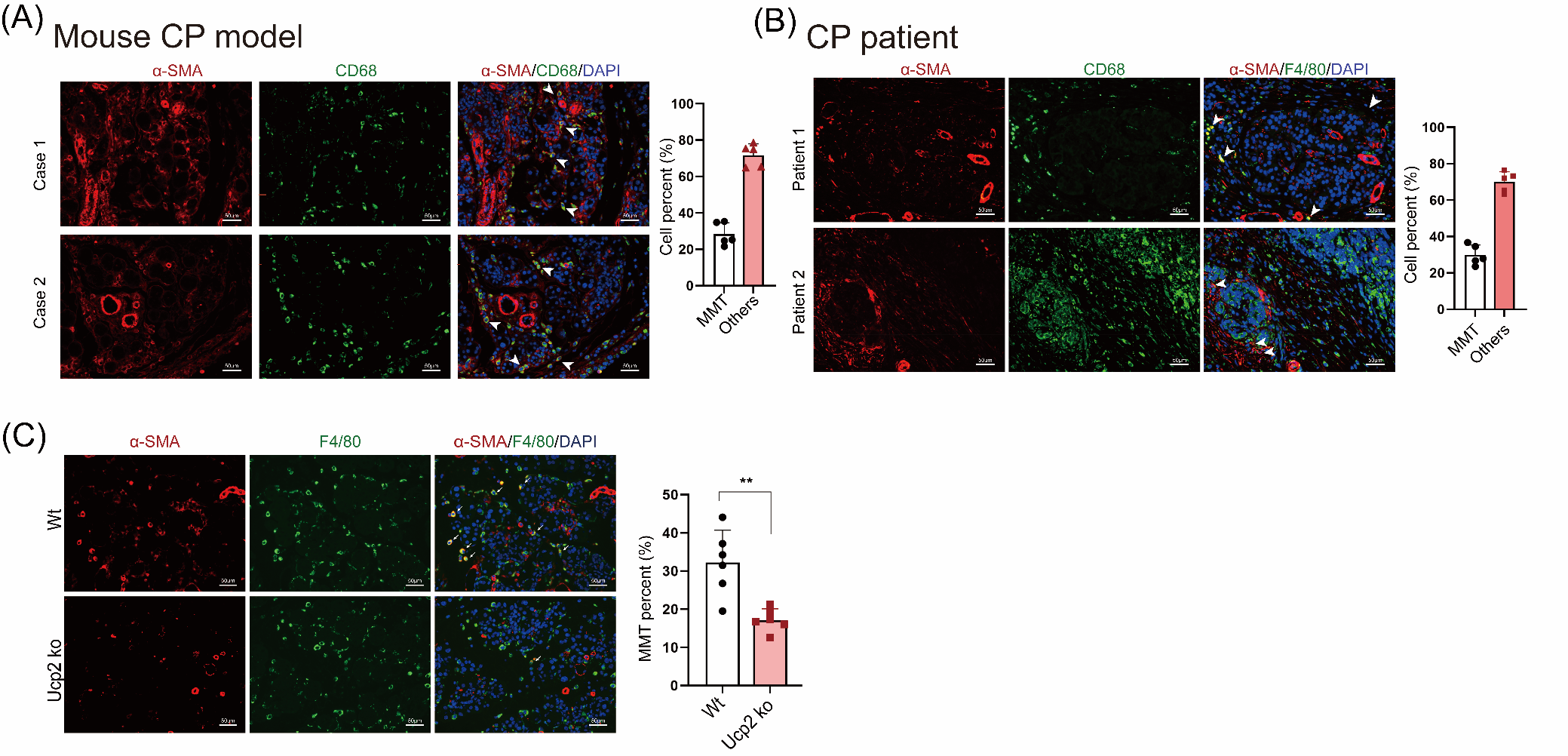


(A) Immunofluorescence analysis of pancreatic tissues from CP mice shows that approximately 30% of α-SMA-positive myofibroblasts co-express macrophage markers F4/80 and CD68, indicating their origin from macrophages. 400X magnification, scale bar, 50 μm. n=5. (B) This finding is consistent with results from CP patient samples. 400X magnification, scale bar,50 μm. n=5.

(C) UCP2 knockout in CP mice significantly reduces fibrosis and inhibits the MMT process in pancreatic tissue (15% vs 30%). n = 5. 400X magnification, scale bar, 50 μm. n=5.

Figure S4. Supplementary WB results related to LD.


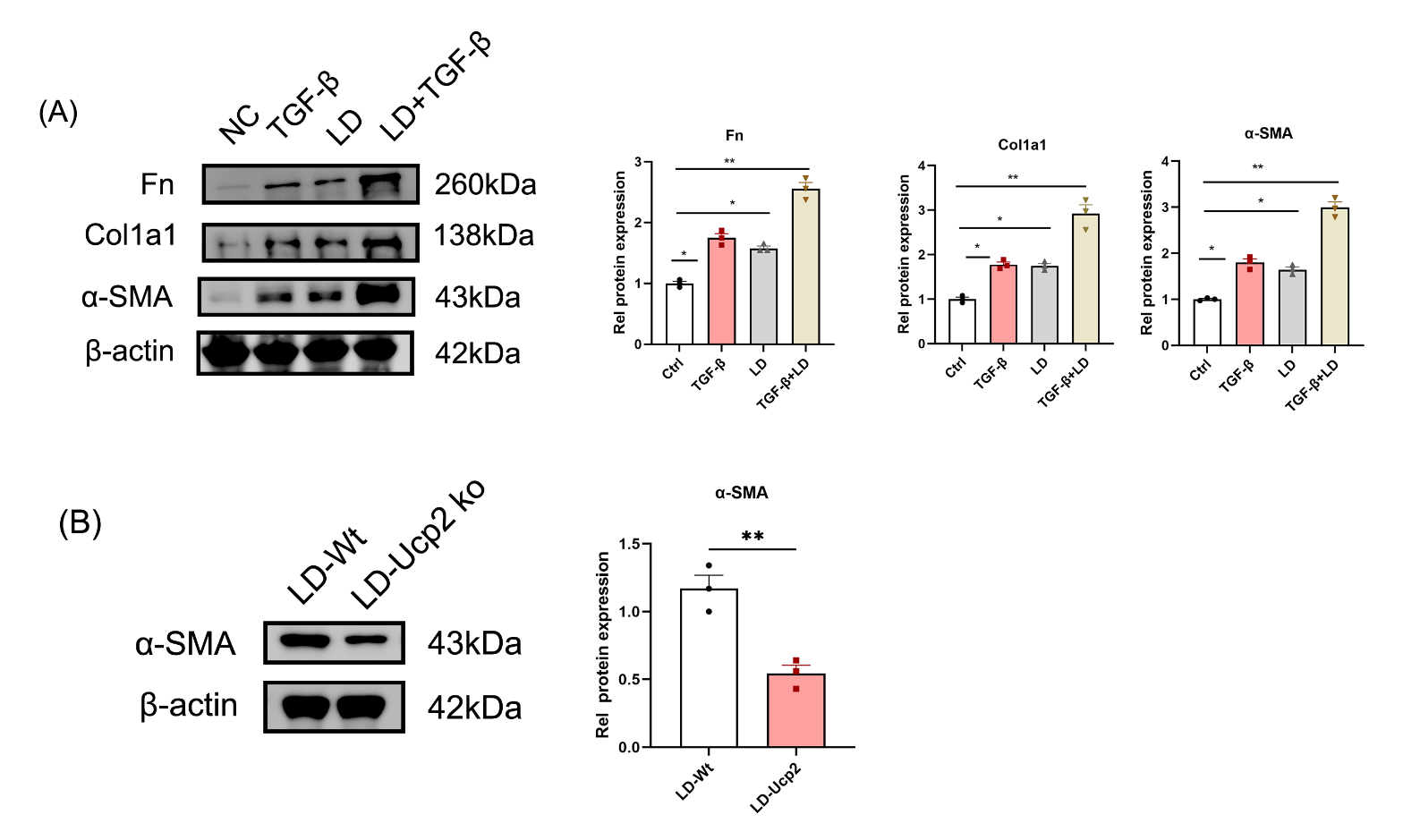


(A) comparable to TGF-β stimulation. Combined stimulation with TGF-β and LD significantly increased protein levels of Fn, Col1a1, and, α-SMA and in BMDMs.n=3. (B)When LD from acinar cells of WT-CP and UCP2 KO-CP mice were co-cultured with BMDMs, UCP2 knockout significantly inhibited MMT in BMDMs.n=3. Data are expressed as the mean ± SEM of three independent experiments; **P* < 0.05, ** *P* < 0.01.

Figure S5. Supplementary Immunohistochemistry results are related to Figure 8B.


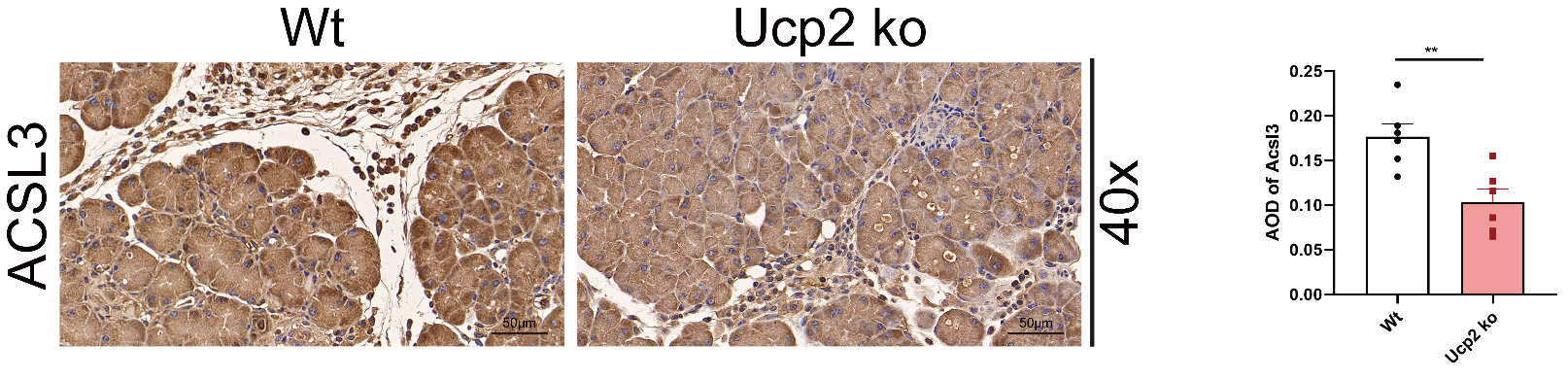


The Immunohistochemistry results corresponding to Figure 8B immunofluorescence show the expression levels of ACSL3 in pancreatic samples from WT and UCP2 KO mice. The results indicate that UCP2 knockout downregulates ACSL3 expression in pancreatic tissue. 400X magnification, scale bar, 50μm.

Figure S6. Supplementary WB results are related to Figure 9.


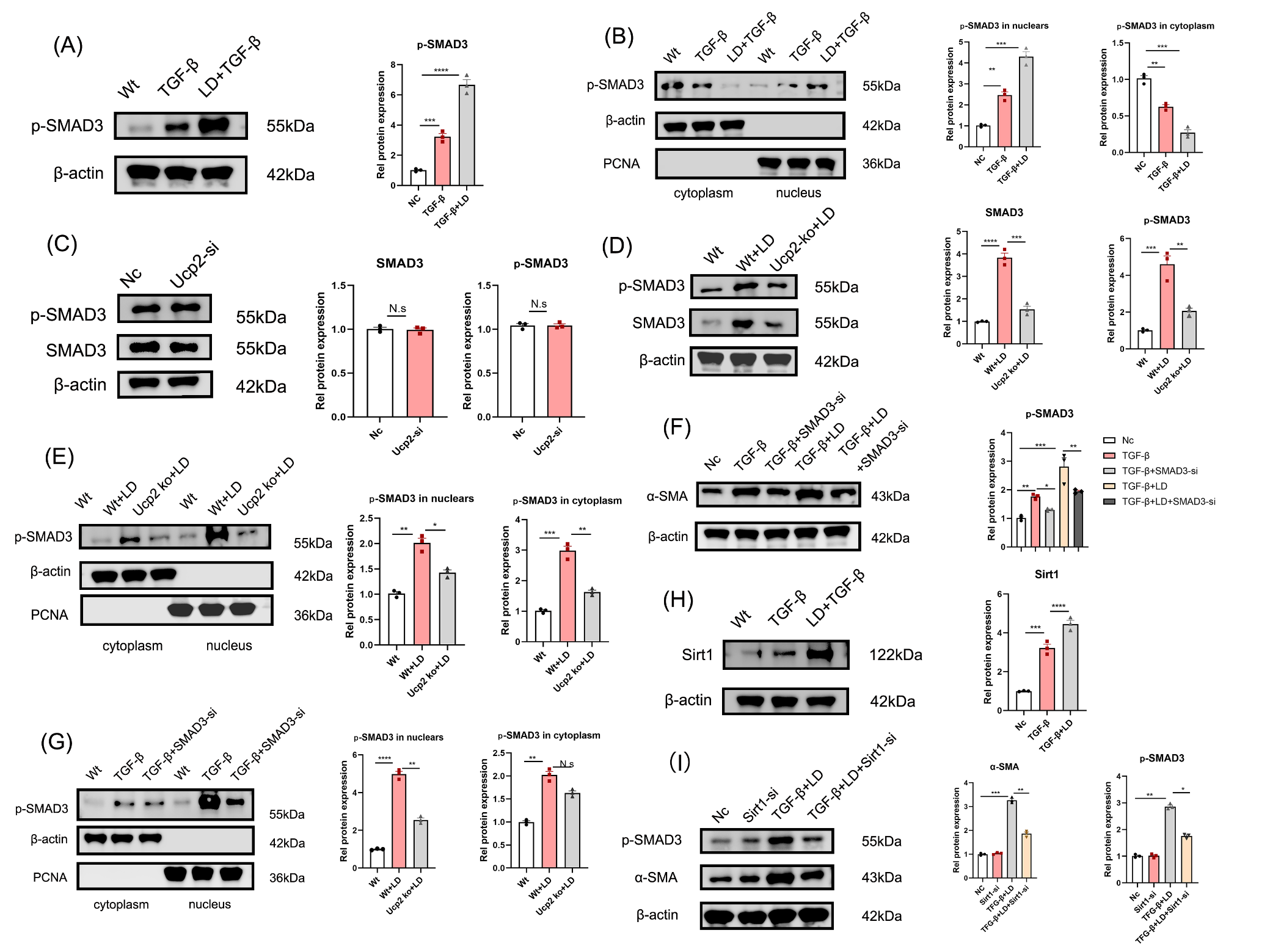


(A-B) TGF-β and LD significantly increased Smad3 expression and nuclear translocation in macrophages (C) UCP2 knockout did not affect baseline Smad3 or p- Smad3 levels in BMDMs. (D-E) UCP2 knockout inhibited TGF-β and lipid droplet-induced Smad3 expression and nuclear translocation. (F-G) Smad3 knockdown reduced α-SMA expression in TGF-β and lipid droplet-induced MMT cells(H) TGF-β upregulated Sirt1 mRNA levels, enhanced by LD. (I) Sirt1 knockdown counteracted TGF-β-induced Smad3 upregulation, while overexpression enhanced it. Data are expressed as the mean ± SEM of at least three independent experiments; **P* < 0.05, ** *P* < 0.01, ****P* < 0.001, *****P* < 0.0001. NS, no significant difference.

Figure S7. Macrophage-specific knockout of Sirt1 inhibits MMT and pancreatic fibrosis.


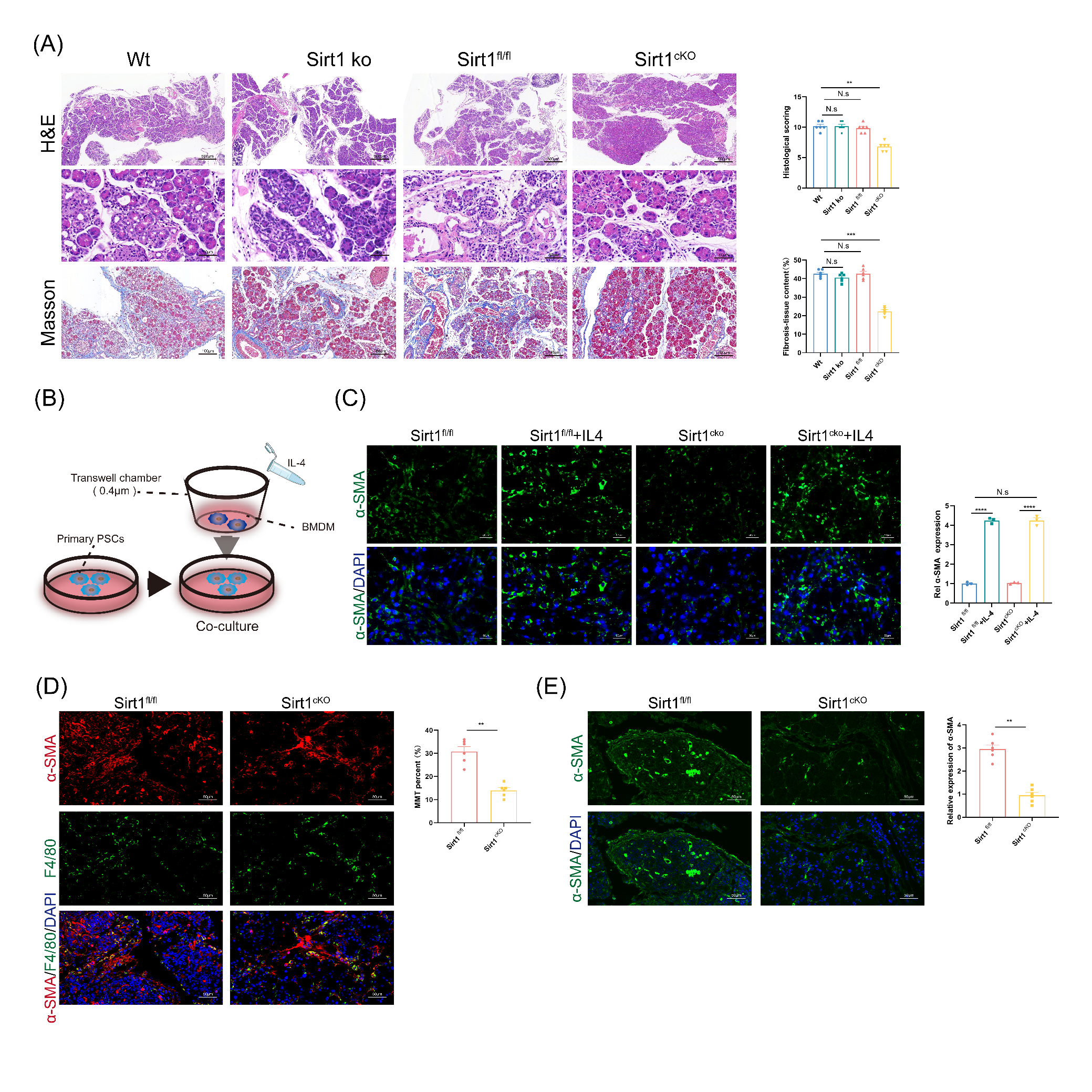


(A) HE staining showed that Sirt1 cKO alleviated the fibrotic characteristics of CP and reduced the histological score .40X or 400X magnification, scale bar, 500 μm or 50 μm. n=6. Masson staining confirmed that Sirt1 cKO alleviated CP-associated fibrosis. 200X magnification, scale bar, 100 μm n=6. (B) Schematic diagram of co-culture. (C) Sirt1 cKO did not alter the fibrotic phenotype of macrophages or PSCs. 400X magnification, scale bar 50 μm.n=3. (D) Sirt1 cKO inhibited the MMT process. 400X magnification, scale bar 50 μm.n=6. (E) α-SMA staining confirmed the inhibition of the MMT process in Sirt1 cKO mice. 400X magnification, scale bar 50 μm.n=6. ** *P* < 0.01, ****P* < 0.001, *****P* < 0.0001. NS, no significant difference.n=3.

References

(1) He, J.; Ma, M.; Li, D.; Wang, K.; Wang, Q.; Li, Q.; He, H.; Zhou, Y.; Li, Q.; Hou, X.; Yang, L. Sulfiredoxin-1 Attenuates Injury and Inflammation in Acute Pancreatitis through the ROS/ER Stress/Cathepsin B Axis. *Cell Death Dis* **2021**, *12* (7), 626. https://doi.org/10.1038/s41419-021-03923-1.

(2) Nadella, S.; Ciofoaia, V.; Cao, H.; Kallakury, B.; Tucker, R. D.; Smith, J. P. Cholecystokinin Receptor Antagonist Therapy Decreases Inflammation and Fibrosis in Chronic Pancreatitis. *Dig Dis Sci* **2020**, *65* (5), 1376–1384. https://doi.org/10.1007/s10620-019-05863-5.

(3) Ammann, R. W.; Heitz, P. U.; Klöppel, G. Course of Alcoholic Chronic Pancreatitis: A Prospective Clinicomorphological Long-Term Study. *Gastroenterology* **1996**, *111* (1), 224–231. https://doi.org/10.1053/gast.1996.v111.pm8698203.
